# GRACE-YOLOv7: An Improved Deep Learning Method for Object Detection in Remote Sensing Images

**DOI:** 10.3390/s26217092

**Published:** 2026-11-09

**Authors:** Xiang Gao, Changbao Yang, Liguo Han

**Affiliations:** College of Geoexploration Science and Technology, Jilin University, Changchun 130026, China

**Keywords:** remote sensing, multi scale, object detection, deformable convolution, attention mechanism

## Abstract

**Highlights:**

- GRACE-YOLOv7 is developed for multi-scale object detection in complex remote sensing scenes.
- The proposed framework jointly enhances spatial feature extraction, multi-scale feature interaction, high-resolution object detection, and bounding-box localization.

**What are the main findings?**

- GRACE-YOLOv7 achieves a mAP0.5 of 86.09% and a mAP0.75 of 65.42%, outperforming YOLOv7 by 2.35 and 3.28 percentage points, respectively.
- The proposed components exhibit complementary effects, with ELAN-D and BRA contributing to improved feature representation and multi-scale feature interaction; the high-resolution detection branch notably improving Recall, and MPDIoU mainly enhancing bounding-box localization.
- GRACE-YOLOv7 improves detection completeness for small and densely distributed objects and achieves better localization performance in complex remote sensing scenes.

**What are the implications of the main findings?**

- Joint improvements in adaptive spatial feature extraction, region-aware feature interaction, high-resolution detection, and bounding-box regression provide an effective strategy for improving multi-scale remote sensing object detection.
- GRACE-YOLOv7 provides a promising approach for reliable object detection in complex remote sensing imagery.

**Abstract:**

The substantial variation in object scales, complex spatial distributions, and rich background information in remote sensing images can lead to inadequate feature representation, missed detections, and inaccurate localization of small and densely distributed objects. To address these issues, this study proposes GRACE-YOLOv7, an improved network for multi-scale object detection in remote sensing images based on YOLOv7. First, an ELAN-D module incorporating deformable convolution v3 (DCNv3) is constructed during deep feature extraction in the backbone. By adaptively adjusting the spatial sampling locations, the proposed module enhances the network’s ability to represent objects with different scales and geometric structures. Second, a Bi-level Routing Attention (BRA) mechanism is introduced at key feature interaction positions in the neck to strengthen information interaction among target-relevant regions and reduce the interference of complex background information with multi-scale feature fusion. On this basis, shallow high-resolution features are incorporated and an additional detection branch is introduced, extending the original three-scale detection structure to a four-scale detection structure to enhance the representation of small and densely distributed objects. Meanwhile, MPDIoU is adopted to replace the original CIoU as the bounding box regression loss to further improve object localization. Experimental results on the DOTA-v1.0 dataset show that GRACE-YOLOv7 achieves mAP0.5, mAP0.75, Recall, Precision, and F1 scores of 86.09%, 65.42%, 84.40%, 82.60%, and 0.8347, respectively. Compared with YOLOv7, mAP0.5 and mAP0.75 increase by 2.35 and 3.28 percentage points, respectively, while Recall and Precision increase by 2.81 and 1.63 percentage points, respectively, and F1 increases from 0.8093 to 0.8347. These results demonstrate that GRACE-YOLOv7 improves multi-scale object detection performance on the DOTA-v1.0 dataset, particularly for small and densely distributed objects.

## 1. Introduction

Remote sensing image object detection is an important research topic in intelligent interpretation of remote sensing imagery. Its primary task is to identify the classes and spatial locations of objects of interest in complex remote sensing scenes [1], and it has received extensive attention in recent years [2]. Accurate object detection can provide essential data support and technical references for urban planning, land-use surveys, traffic monitoring, disaster assessment, ecological and environmental protection, and military reconnaissance [3,4]. In recent years, the rapid development of aerial remote sensing platforms, unmanned aerial vehicle systems, and high-resolution imaging technologies has substantially enhanced the capacity to acquire remote sensing imagery, with high-spatial-resolution images exhibiting significant improvements in spatial details, land-cover information, and data volume [5]. Meanwhile, the growing demand for the automated processing of massive volumes of remote sensing imagery has placed higher requirements on the accuracy and robustness of object detection algorithms [6]. Compared with natural scene images, remote sensing imagery is generally characterized by substantial variations in object scales, dense spatial distributions, and complex background textures [3,7]. Particularly in high-spatial-resolution remote sensing imagery, many objects occupy only a limited number of pixels, resulting in pronounced scale differences across object categories. Moreover, objects within the same category may exhibit substantial variations in appearance due to differences in imaging scale, viewing angle, and occlusion [2,8]. Therefore, how to effectively extract and fuse informative features of objects at different scales in complex backgrounds, while improving the detection and localization accuracy of small and densely distributed objects, has become an important research issue in remote sensing object detection.

In recent years, advances in deep learning have significantly promoted the development of object detection techniques. Depending on the detection paradigm, existing deep learning-based object detection methods can be broadly categorized into two-stage and single-stage detectors. Two-stage methods, represented by the R-CNN family, perform object detection through region proposal generation followed by object classification and bounding box regression [9]. Building on this framework, methods such as Fast R-CNN and Faster R-CNN further improved detection accuracy and localization capability [10,11]. However, two-stage detectors generally require additional region proposal generation and processing, resulting in relatively complex network architectures and detection procedures as well as substantial computational costs, which limits their application in scenarios with stringent requirements for detection efficiency [12,13]. In contrast, single-stage detection methods, represented by SSD, RetinaNet, and the YOLO series, directly perform object classification and bounding box regression within a unified network, eliminating the separate region proposal generation process and achieving a favorable balance between detection accuracy and inference efficiency [14,15,16]. Among these methods, SSD performs object prediction using feature maps at different scales; however, the relatively limited semantic information in shallow features results in insufficient representation of small objects [17]. RetinaNet alleviates the imbalance between foreground and background samples through Focal Loss, but its multi-scale feature representation in complex remote sensing scenes still has room for improvement [18]. To improve the representation of objects at different scales, multi-scale feature fusion has gradually become an important component of object detection networks. Feature Pyramid Networks enhance the representation of multi-scale objects by integrating semantic and spatial information from different feature levels [18]. Meanwhile, attention mechanisms can adaptively adjust feature responses across different channels or spatial locations, highlighting target-relevant information and reducing the influence of redundant features, thereby providing a new approach for enhancing target features in complex scenes [19,20].

The YOLO series, as a representative class of one-stage object detection methods, integrates object classification and bounding box regression into an end-to-end network framework and achieves a favorable balance between detection speed and accuracy. It has therefore been widely applied to remote sensing object detection tasks [16]. Among these methods, YOLOv7 achieves a favorable balance among detection accuracy, inference speed, and model complexity through optimized network architecture and training strategies, providing a solid foundation for developing efficient remote sensing object detection models [21]. However, YOLOv7 was originally designed for general-purpose object detection and still has room for further optimization when directly applied to multi-scale object detection in remote sensing imagery. First, small objects in remote sensing imagery typically contain only a limited number of effective pixels. As the network undergoes successive downsampling and deep feature extraction, some spatial details in shallow features can gradually be weakened, thereby affecting the effective representation of small and densely distributed objects [22]. Second, standard convolution employs fixed and regular sampling locations. When objects vary in scale, aspect ratio, and spatial structure, this fixed local sampling scheme cannot adaptively adjust to object-specific characteristics, thereby limiting the ability of feature extraction to accommodate diverse spatial structures [23]. Third, remote sensing imagery typically covers large geographic areas and contains complex background compositions. During multi-scale feature propagation and fusion, background information unrelated to the target may be propagated together with the target features, thereby weakening the network’s ability to focus on key target regions and relevant contextual information [24]. In addition, in scenes containing small, densely distributed, and substantially varying-scale objects, deviations may occur in the positions and dimensions of predicted bounding boxes, indicating the need for further optimization of the bounding box regression process. Therefore, YOLOv7 requires targeted improvements for multi-scale object detection in remote sensing imagery, particularly in terms of preserving high-resolution details, adaptive spatial feature extraction, effective information interaction in complex backgrounds, and accurate bounding box localization.

Recent studies have addressed these challenges through complementary architectural strategies. Shin et al. proposed DCEF^2^-YOLO, which combines deformable convolution with an efficient feature-fusion structure for aerial small-target detection [25]. Zhao et al. improved YOLOv7 by reducing information loss during feature transmission, introducing an attention mechanism and additional cross-layer connections, and modifying the training strategy [26]. Chen et al. developed DB-YOLO by integrating bi-level routing attention with DCNv2 and using an NWD-CIoU localization loss [27]. These studies establish close precedents for deformable sampling, attention-based feature interaction, and improved use of small-object information. GRACE-YOLOv7 differs in the deformable-convolution variant and its placement, the integration of BRA into the neck, and the detection topology: it incorporates DCNv3 in ELAN-D, uses Up-BRA and MP-BRA for region-aware feature processing in the neck, and extends the original three-scale detector with a fourth, high-resolution branch. It also adopts MPDIoU for box regression. Thus, the proposed architecture combines these components in a YOLOv7-based design tailored to multi-scale remote-sensing detection; it does not claim the underlying DCNv3, BRA, or MPDIoU mechanisms as new.

To address the above issues, this study proposes GRACE-YOLOv7 (Geometry- and Routing-Aware Cross-scale Enhancement YOLOv7), an improved network for multi-scale object detection in remote sensing imagery based on YOLOv7. The underlying DCNv3 operator [24], Bi-level Routing Attention (BRA) [28], and MPDIoU are adopted from existing work. The architectural contributions lie in their task-specific integration and in the added cross-scale detection design: (1) ELAN-D adapts the existing ELAN structure by incorporating DCNv3 in deep feature extraction; (2) Up-BRA and MP-BRA place BRA-based region interaction at selected neck stages to guide top-down and bottom-up feature processing; and (3) a shallow high-resolution feature path and an additional detection head extend YOLOv7 from three detection scales to four. MPDIoU replaces the baseline CIoU as the adopted localization loss rather than a newly proposed loss. Compared with DCEF2-YOLO, the present design uses DCNv3 within ELAN-D and introduces a fourth detection scale; compared with the YOLOv7 modifications of Zhao et al., it centers on DCNv3-based spatial adaptation and BRA-guided neck fusion; and compared with DB-YOLO, it uses DCNv3 instead of DCNv2, locates BRA in the neck rather than the ELAN module, adds a high-resolution detection branch, and adopts MPDIoU instead of NWD-CIoU. These changes are intended to improve adaptive spatial representation, multi-scale feature interaction, and detection of small and densely distributed objects.

The remainder of this paper is organized as follows. Section 2 introduces the overall network architecture of GRACE-YOLOv7 and its improved modules. Section 3 describes the experimental data, data preprocessing, experimental settings, evaluation metrics, and comparison methods. Section 4 analyzes and discusses the model detection performance, ablation experiments, and comparative results with different object detection methods. Section 5 summarizes the main conclusions of this study.

## 2. Methods

### 2.1. Overall Architecture of the Proposed GRACE-YOLOv7

**Figure 1:**
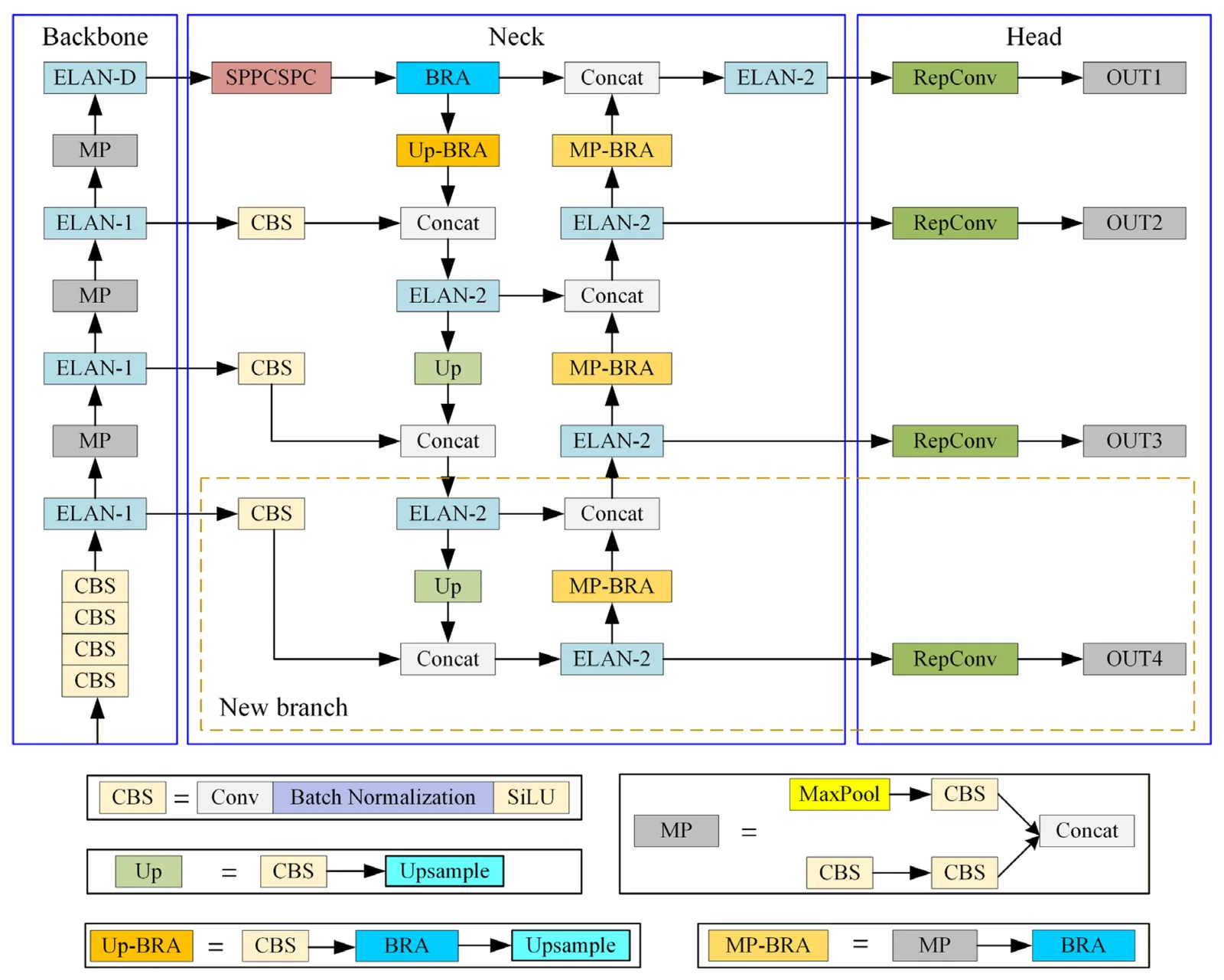
Overall architecture of the proposed GRACE-YOLOv7.

To address the challenges of complex backgrounds, substantial object-scale variations, and difficult detection of small and densely distributed objects in remote sensing imagery, an improved object detection network, GRACE-YOLOv7 (Geometry- and Routing-Aware Cross-scale Enhancement YOLOv7), is developed based on YOLOv7. Its overall architecture is illustrated in Figure 1. The proposed model mainly consists of four components: Input, Backbone, Neck, and Head. While retaining the basic detection framework of YOLOv7, the network is improved in terms of deep feature extraction, multi-scale feature fusion, and high-resolution object detection. Specifically, ELAN-D is introduced into the Backbone to enhance the adaptability of high-level features to variations in object geometry. BRA is incorporated at key feature interaction locations in the Neck, together with the proposed Up-BRA and MP-BRA modules, to strengthen information interaction among target-related regions and features at different scales. In addition, shallow high-resolution features are further incorporated into the feature fusion network, resulting in a four-scale detection structure in the Head to enhance the representation of small and densely distributed objects. During training, MPDIoU is employed to replace the original CIoU for bounding-box regression optimization. It should be clarified that DCNv3, BRA, and MPDIoU are adopted from existing studies, whereas the architectural contributions of this work lie in the localized DCNv3-based ELAN-D design, the selective deployment of BRA through the proposed Up-BRA and MP-BRA modules, the introduction of an additional high-resolution detection branch, and the integration of these modifications within the GRACE-YOLOv7 framework.

#### 2.1.1. Input

The input images are resized to 640 × 640 pixels before being fed into the network. The input features first pass through a sequence of CBS modules for preliminary feature extraction and spatial resolution adjustment, and are then progressively encoded by the Backbone. The CBS module consists of a convolution operation, Batch Normalization, and SiLU activation, as illustrated in Figure 1.

#### 2.1.2. Backbone

**Figure 2:**
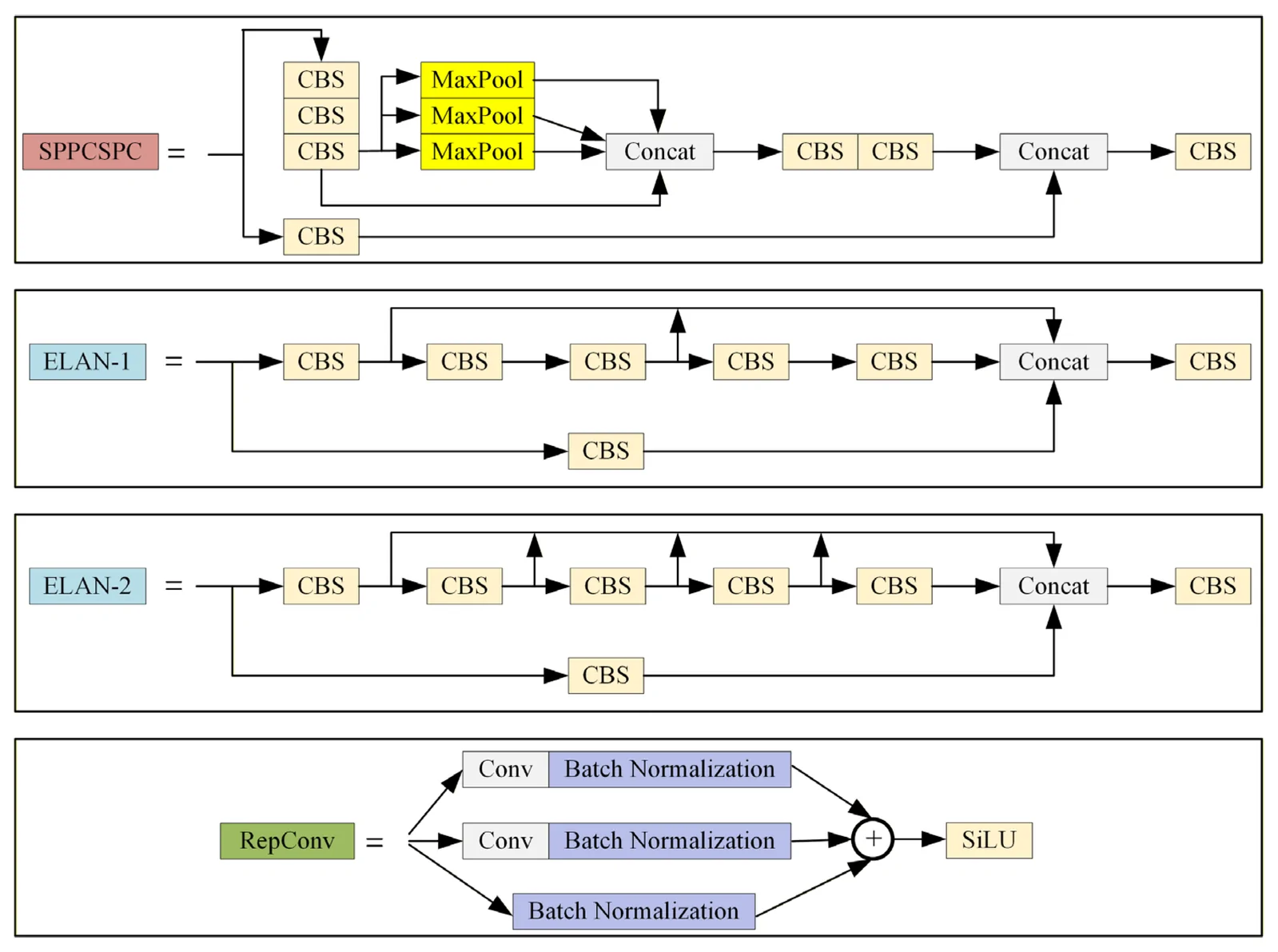
Structures of the main modules in GRACE-YOLOv7.

The Backbone mainly consists of CBS, ELAN-1, MP, and ELAN-D modules, which progressively extract hierarchical spatial and semantic features through successive downsampling and multi-branch feature aggregation. After passing through the initial CBS modules, the features are sequentially processed by multiple ELAN-1 and MP modules for hierarchical feature extraction. ELAN-1 enhances information transmission among features of different depths through multi-branch convolutional paths and feature concatenation, as shown in Figure 2, while the MP module performs spatial downsampling and feature aggregation through parallel branches, as illustrated in Figure 1.

At the deep feature extraction stage of the Backbone, the last ELAN module is modified into ELAN-D. While retaining the original multi-branch connections and feature aggregation scheme, ELAN-D incorporates CBS-D constructed with DCNv3, enabling the deep features to adaptively adjust their sampling locations according to object scale, shape, and spatial structure. This improves the spatial representation capability of high-level semantic features. The detailed structures of ELAN-D and CBS-D are presented in Section 2.3.

#### 2.1.3. Neck

The Neck is responsible for bidirectional fusion of multi-level features extracted by the Backbone, thereby integrating high-level semantic information with shallow spatial details. As shown in Figure 1, the deep features from the Backbone are first processed by SPPCSPC for multi-scale contextual aggregation, after which BRA is introduced to model the correlations among different spatial regions, allowing subsequent feature fusion to focus more strongly on highly relevant target regions.

During top-down feature propagation, the highest-level features are first processed by the Up-BRA module. Up-BRA sequentially consists of CBS, BRA, and Upsample, enabling region-aware feature enhancement before increasing the spatial resolution. The resulting features are then concatenated with the corresponding Backbone features and further aggregated by ELAN-2. The remaining upsampling stages employ conventional up modules, progressively integrating shallow spatial details with deep semantic information through upsampling and feature concatenation.

To further exploit the rich spatial information contained in shallow features, the feature fusion process is extended to a higher-resolution level, expanding the original three-scale feature fusion structure into a four-scale structure. The high-resolution features from the shallow stage of the Backbone are first processed by CBS for channel adjustment and then concatenated with progressively upsampled deep features. The concatenated features are subsequently processed by ELAN-2 to generate high-resolution fused features. These features are used not only for the subsequent high-resolution detection branch but also for continued bottom-up multi-scale feature fusion.

During bottom-up feature propagation, the conventional MP unit is extended to the proposed MP-BRA module. MP first performs spatial downsampling and feature aggregation, after which BRA selectively enhances spatially relevant information within the fused features. Through repeated downsampling, feature concatenation, and ELAN-2 aggregation, sufficient interaction among features of different resolutions is achieved. The structures of the corresponding local modules are illustrated in Figure 1 and Figure 2.

#### 2.1.4. Head

The Head receives four feature maps with different spatial resolutions from the Neck and performs object prediction at each scale. For an input size of 640 × 640 pixels, the four detection branches correspond to feature map resolutions of 160 × 160, 80 × 80, 40 × 40, and 20 × 20, respectively. Among them, the 160 × 160 high-resolution feature map integrates shallow spatial details from the Backbone with high-level semantic information propagated through the Neck, making it more effective for preserving local spatial characteristics of small and densely distributed objects. The detection branches at different resolutions jointly provide multi-scale feature representation and prediction.

The fused features at each scale are first processed by RepConv and subsequently fed into the corresponding prediction layers to generate object location, category, and confidence information. During training, RepConv consists of three parallel branches: 3 × 3 convolution with Batch Normalization, 1 × 1 convolution with Batch Normalization, and identity mapping with Batch Normalization. The outputs of the three branches are summed and then passed through a SiLU activation function, as shown in Figure 2. By introducing the additional high-resolution detection branch, the proposed model makes greater use of shallow spatial details while retaining multi-scale semantic information, thereby improving the representation capability for small objects, densely distributed objects, and objects with substantial scale variations in remote sensing imagery.

### 2.2. BRA-Enhanced Feature Fusion Module

Remote sensing images are generally characterized by large scene coverage, complex background composition, and substantial variations in object scale and spatial distribution. Background information can easily propagate together with target features during multi-scale feature fusion, thereby interfering with the representation of effective target information. YOLOv7 mainly realizes information interaction among features at different scales through convolution operations and feature concatenation, but lacks explicit modeling of the relationships among different spatial regions. Therefore, Bi-level Routing Attention (BRA) [29] is introduced to enhance spatial information interaction during multi-scale feature fusion.

**Figure 3:**
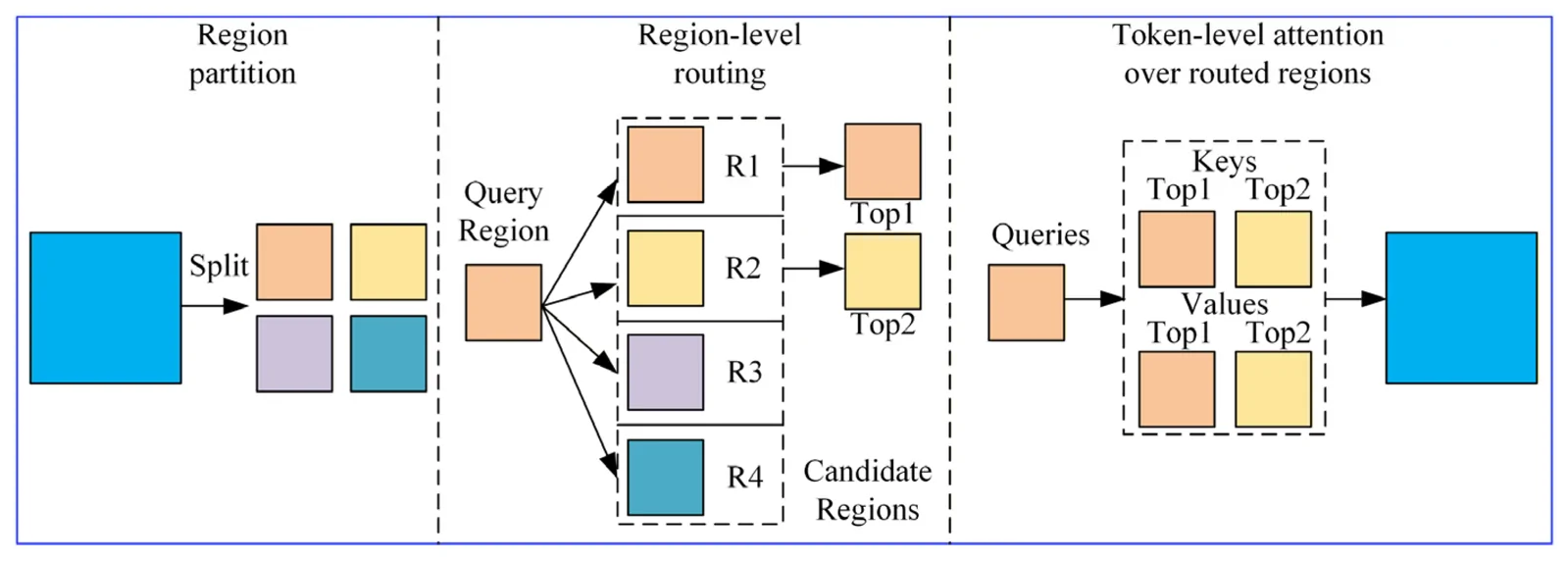
Structure of the Bi-level Routing Attention Module.

As shown in Figure 3, BRA adopts a coarse-to-fine bi-level attention mechanism. First, the input feature map is partitioned into several non-overlapping local regions, and the features within each region are aggregated to obtain region-level representations. On this basis, the relevance between the current query region and the remaining regions is calculated, and the Top-k most relevant regions are selected for each query region through dynamic routing. This process first performs information filtering at the region level, allowing subsequent feature interactions to focus on regions with higher semantic relevance. In this way, redundant interactions among irrelevant regions are reduced while the capability of modeling long-range dependencies across regions is retained. After region-level routing, BRA further performs fine-grained attention within the selected Top-k routed regions. Features from the current query region serve as the Query, while the corresponding Key and Value features are obtained from the selected relevant regions, thereby establishing more fine-grained feature dependencies.

The above coarse-to-fine routing process can be further formulated mathematically as follows. Given an input feature map $X\in R^{H\times W\times C}$, it is first divided into S×S non-overlapping regions:

(1) $$X\to {\left\{X_{i}\right\}}_{i=1}^{N_{r}},\;N_{r}=S^{2},$$

where $N_{r}$ denotes the number of regions. In the present implementation, S=7, resulting in 49 spatial regions. The Query, Key, and Value features are generated from the input features through linear projections:

(2) $$Q=XW_{Q},\;K=XW_{K},\;V=XW_{V},$$

where $W_{Q}$, $W_{K}$, and $W_{V}$ denote the corresponding learnable projection matrices. For the *i*-th region, the region-level Query and Key representations are obtained by averaging the token features within the region:

(3) $${\bar{q}}_{i}=\frac{1}{M}\sum_{t=1}^{M}q_{i,t},\;{\bar{k}}_{i}=\frac{1}{M}\sum_{t=1}^{M}k_{i,t},$$

where M denotes the number of tokens contained in each region.

The relevance between the *i*-th query region and the *j*-th candidate region is then calculated using scaled dot-product similarity:

(4) $$R_{ij}=\frac{{\bar{q}}_{i}{\bar{k}}_{j}^{T}}{\sqrt{d_{qk}}},$$

where $d_{qk}$ denotes the feature dimension used for Query-Key matching. For each query region, only the k regions with the highest relevance scores are retained through Top-k routing:

(5) $$I_{i}=\mathrm{TopK}\left(R_{i,:},k\right)$$

In this study, k is fixed to 4 for all BRA modules. Therefore, each query region selectively interacts with the four most relevant regions rather than all spatial regions, which reduces unnecessary interactions with weakly related or background regions.

According to the routing index $I_{i}$, the corresponding Key and Value tokens from the selected regions are gathered to form $K_{i}^{r}$ and $V_{i}^{r}$. Fine-grained attention is subsequently performed between the Query tokens of the current region and the routed Key-Value features:

(6) $$A_{i}=\mathrm{Softmax}\left(\frac{Q_{i}{\left(K_{i}^{r}\right)}^{T}}{\sqrt{d_{h}}}\right),$$

(7) $$Z_{i}=A_{i}V_{i}^{r},$$

where $d_{h}$ denotes the feature dimension of each attention head. Through this process, BRA first determines the most relevant spatial regions at a coarse level and subsequently establishes token-level feature dependencies only within the selected regions. This coarse-to-fine strategy enables the network to retain long-range contextual information while avoiding redundant global feature interactions.

Through the two-level structure of coarse region-level routing and fine-grained interaction within routed regions, BRA can adaptively focus on relevant spatial regions according to the input content, while maintaining both computational efficiency and cross-region feature modeling capability.

In the proposed method, BRA is selectively deployed at key feature interaction locations in the Neck to enhance region selection and contextual modeling during multi-scale feature fusion. For remote sensing scenes with complex backgrounds, substantial scale variations, and densely distributed objects, region-level dynamic routing helps reduce interference from irrelevant background regions, while subsequent fine-grained attention further strengthens local and cross-region information related to the targets. Therefore, the introduction of BRA can improve the network’s ability to select effective target features under complex backgrounds and enhance contextual representations for dense and multi-scale objects.

### 2.3. DCNv3-Enhanced ELAN-D Module

Objects in remote sensing images often exhibit substantial variations in scale, shape, and spatial structure. In contrast, standard convolution adopts a fixed and regular sampling pattern whose sampling locations do not change according to object geometry. Therefore, its adaptability to different spatial structures of objects can still be further improved. To enhance the feature extraction capability of the model for objects with diverse geometric structures, Deformable Convolution v3 (DCNv3) is introduced into the deep feature extraction stage of YOLOv7 [30].

**Figure 4:**
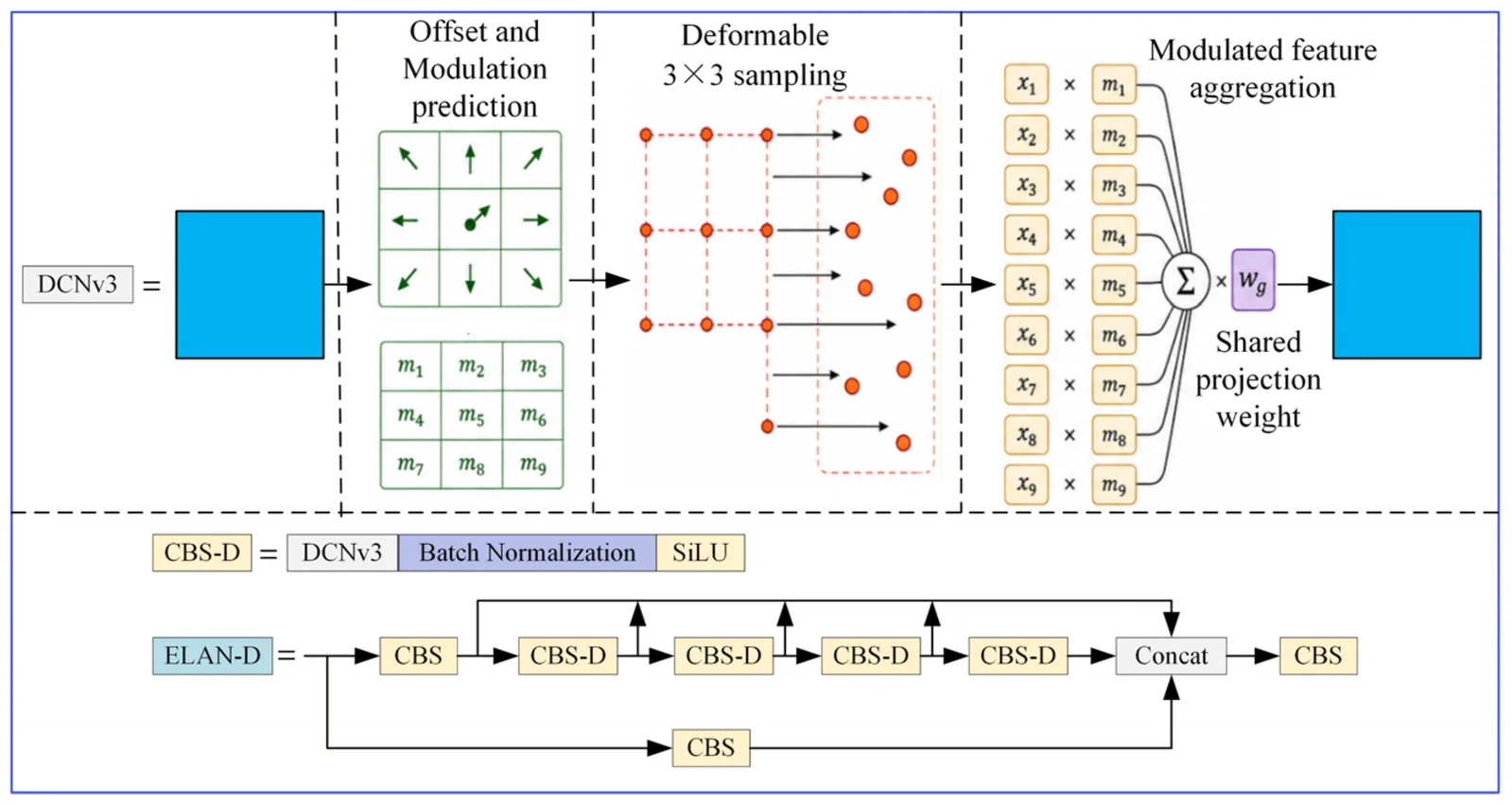
Structures of the DCNv3, CBS-D, and ELAN-D modules.

As shown in Figure 4, DCNv3 dynamically predicts spatial offsets and modulation weights for convolutional sampling points according to the input features, allowing the originally regular sampling locations to adapt to local object structures. Meanwhile, the contribution of different sampled features to the final feature aggregation is adaptively adjusted. Specifically, the spatial offsets determine where the features are sampled, while the modulation weights control the relative importance of different sampling locations. For sampling points shifted to non-integer coordinates, the corresponding feature responses are obtained through bilinear interpolation. In addition, DCNv3 adopts a grouped spatial aggregation mechanism that divides the input channels into multiple feature groups, enabling different groups to learn distinct spatial sampling patterns and thereby improving the adaptability of different feature subspaces to local structural variations in objects. 

The above adaptive sampling and feature aggregation process can be further formulated mathematically as follows. Given an input feature map *X*, a local feature representation used for predicting the sampling parameters is first generated as

(8) F=ϕ(X),

where ϕ(·) denotes the feature transformation used to generate the spatially adaptive parameters. The spatial offsets and unnormalized modulation coefficients are then predicted from F through learnable transformations:

(9) $$\Delta P=W_{\Delta }F+b_{\Delta },\;\hat{M}=W_{m}F+b_{m},$$

where ΔP contains the two-dimensional spatial offsets and $\hat{M}$ contains the corresponding modulation coefficients. For the *g*-th feature group and the *k*-th sampling point, the predicted quantities are denoted as $\Delta p_{gk}$ and ${\hat{m}}_{gk}$, respectively.

To stabilize the contribution of different sampling points, DCNv3 normalizes the modulation coefficients within each feature group using the Softmax function:

(10) $$m_{gk}=\frac{\exp\left({\hat{m}}_{gk}\right)}{\sum_{j=1}^{K}\exp\left({\hat{m}}_{gj}\right)},$$

where K denotes the number of sampling points. Thus,

(11) $$\sum_{k=1}^{K}m_{gk}=1.$$

The deformable feature aggregation of DCNv3 can then be expressed as

(12) $$y\left(p_{0}\right)=\sum_{g=1}^{G}\sum_{k=1}^{K}w_{g}m_{gk}x_{g}\left(p_{0}+p_{k}+\Delta p_{gk}\right),$$

where $p_{0}$ denotes the current spatial location, G is the number of feature groups, $x_{g}$ represents the input feature of the *g*-th group, $p_{k}$ is the predefined regular sampling position, $\Delta p_{gk}$ is the learned offset of the *k*-th sampling point in the *g*-th group, $m_{gk}$ is its normalized modulation weight, and $w_{g}$ denotes the shared projection weight associated with the *g*-th feature group. When $p_{0}+p_{k}+\Delta p_{gk}$ corresponds to a non-integer coordinate, the feature value is obtained through bilinear interpolation.

Through this mechanism, the sampling offsets enable the convolutional kernel to adaptively change where features are collected according to the local spatial structure, while the modulation weights determine how strongly the sampled features contribute to the aggregation result. Furthermore, different feature groups can learn different spatial sampling patterns. Combined with normalization of the modulation weights, DCNv3 introduces content-adaptive spatial sampling while preserving the local modeling capability of convolution, thereby improving the adaptability of feature extraction to objects with different scales and geometric structures.

The proposed model does not replace all standard convolutions with DCNv3; instead, a localized modification is applied to the high-level semantic feature extraction stage. As shown in Figure 1, the last ELAN module in the Backbone is modified to construct the proposed ELAN-D module. Specifically, only the original 3 × 3 CBS blocks in the deep feature extraction path are replaced by CBS-D, while the original 1 × 1 CBS blocks, multi-branch connections, and feature aggregation structure remain unchanged. As shown in Figure 4, CBS-D consists of DCNv3, Batch Normalization, and SiLU activation. Through this localized replacement strategy, adaptive spatial sampling is introduced into high-level semantic feature extraction while preserving the original feature aggregation structure of ELAN. This design enhances the adaptability of the network to variations in object scale, aspect ratio, and geometric structure, and provides more effective high-level feature representations for subsequent multi-scale feature fusion and remote sensing object detection.

### 2.4. MPDIoU-Based Bounding-Box Regression Loss

The original YOLOv7 employs CIoU for bounding-box regression, which jointly considers the overlap between the predicted and ground-truth boxes, the distance between their center points, and the difference in aspect ratio. However, the shape constraint in CIoU is mainly formulated based on the relative aspect ratio. When the predicted and ground-truth boxes have similar aspect ratios but still differ in their actual width, height, or spatial location, there remains room for improving the geometric characterization of the bounding-box discrepancy. Considering the large-scale variation and dense-object distribution in DOTA-v1.0, a more explicit geometric constraint may contribute to more accurate object localization. Therefore, Minimum Point Distance IoU (MPDIoU), originally proposed by Ma and Xu [29], is adopted in this study to replace CIoU in the bounding-box regression component of YOLOv7.

MPDIoU extends the conventional IoU metric by explicitly incorporating the distances between corresponding corner points of the predicted and ground-truth boxes. Let the predicted box and ground-truth box be represented as follows:

(13) $$B^{p}=(x_{1}^{p},y_{1}^{p},x_{2}^{p},y_{2}^{p})$$

and

(14) $$B^{gt}=(x_{1}^{gt},y_{1}^{gt},x_{2}^{gt},y_{2}^{gt})$$

respectively, where (*x*_1_,*y*_1_) and (*x*_2_,*y*_2_) denote the coordinates of the top-left and bottom-right corners of a bounding box. The squared Euclidean distances between the two pairs of corresponding corners are calculated as follows:

(15) $$d_{1}^{2}={\left(x_{1}^{p}-x_{1}^{gt}\right)}^{2}+{\left(y_{1}^{p}-y_{1}^{gt}\right)}^{2}$$

(16) $$d_{2}^{2}={\left(x_{2}^{p}-x_{2}^{gt}\right)}^{2}+{\left(y_{2}^{p}-y_{2}^{gt}\right)}^{2}$$

Accordingly, MPDIoU is defined as:

(17) $$MPDIoU=IoU-\frac{d_{1}^{2}}{W^{2}+H^{2}}-\frac{d_{2}^{2}}{W^{2}+H^{2}}$$

where *W* and *H* denote the width and height of the input image, respectively, and are used to normalize the corner-point distances. By simultaneously minimizing the distances between the top-left and bottom-right corners of the predicted and ground-truth boxes, MPDIoU imposes a direct geometric constraint on both the position and scale of the predicted bounding box. As the two corresponding corner points approach those of the ground-truth box, the center position, width, and height of the predicted box are jointly adjusted toward the ground truth.

During model training, the MPDIoU-based bounding-box regression loss is defined as follows:

(18) $$L_{\mathrm{MPDIOU}}=1-MPDIOU$$

which can be expanded as follows:

(19) $$L_{\mathrm{MPDIOU}}=1-IoU+\frac{d_{1}^{2}}{W^{2}+H^{2}}+\frac{d_{2}^{2}}{W^{2}+H^{2}}$$

Therefore, the optimization process simultaneously increases the overlap between the predicted and ground-truth boxes and reduces the geometric distances between their corresponding corner points. In the proposed model, MPDIoU is employed only to replace the CIoU-based bounding-box regression loss of the original YOLOv7, while the objectness and classification losses retain their original formulations. The overall training loss can therefore be expressed as follows:

(20) $$L_{\mathrm{total}}=\lambda_{\mathrm{box}}L_{\mathrm{MPDIOU}}+\lambda_{\mathrm{obj}}L_{\mathrm{obj}}+\lambda_{\mathrm{cls}}L_{\mathrm{cls}}$$

where *L_obj_* and *L_cls_* denote the objectness and classification losses, respectively, and $\lambda_{\mathrm{box}}$, $\lambda_{\mathrm{obj}}$ and $\lambda_{\mathrm{cls}}$ are their corresponding weighting coefficients. In this manner, MPDIoU enhances the bounding-box localization component while preserving the original objectness and classification learning mechanisms of YOLOv7.

## 3. Experimental Setup

### 3.1. Dataset and Data Preprocessing

The DOTA-v1.0 dataset was used in this study. It contains 2806 high-resolution aerial images acquired from different sensors and platforms, with image sizes ranging approximately from 800 × 800 to 4000 × 4000 pixels, and includes a total of 188,282 object instances. The dataset contains 15 typical object categories, namely small vehicle (sv), large vehicle (lv), plane (pl), storage tank (st), ship, harbor (har), ground track field (gtf), soccer ball field (sbf), tennis court (tc), swimming pool (sp), baseball diamond (bd), roundabout (ra), basketball court (bc), bridge (b), and helicopter (heli).

In this study, the 1411 training images and 458 validation images with publicly available annotations were combined, resulting in a total of 1869 original images for reconstructing the experimental dataset. The horizontal bounding boxes provided in DOTA Task2 were used as the target annotations. This setting was adopted to maintain consistency with the original YOLOv7 detection framework and to evaluate the proposed architectural improvements under the same horizontal bounding-box detection task. Before image cropping, the 1869 original images were first divided into mutually exclusive training, validation, and test subsets. During this partitioning process, the distributions of the 15 object categories were also considered, so that the proportions of objects in the training, validation, and test sets were kept as close as possible to 70%, 10%, and 20%, respectively. Considering that the original DOTA images are relatively large and difficult to directly input into object detection networks, a sliding-window strategy was then adopted independently within each subset to crop the original images. The window size was set to 1024 × 1024 pixels, with an overlap of 200 pixels between adjacent windows and a corresponding stride of 824 pixels. Image regions at the boundaries that were smaller than 1024 × 1024 pixels were padded, while the coordinates of the target bounding boxes were transformed accordingly. For objects crossing patch boundaries, the clipped bounding box was retained only when at least 70% of the original bounding-box area remained visible; otherwise, the object was discarded. If the same object satisfied this criterion in multiple overlapping patches, it was retained as an independent annotation in each corresponding patch. During testing, NMS was performed within each patch, while no global NMS or cross-patch bounding-box merging was applied because the evaluation was conducted at the patch level. Thus, all cropped patches generated from the same original image remained in the same subset, avoiding overlap from the same source image across the training, validation, and test sets.

**Figure 5:**
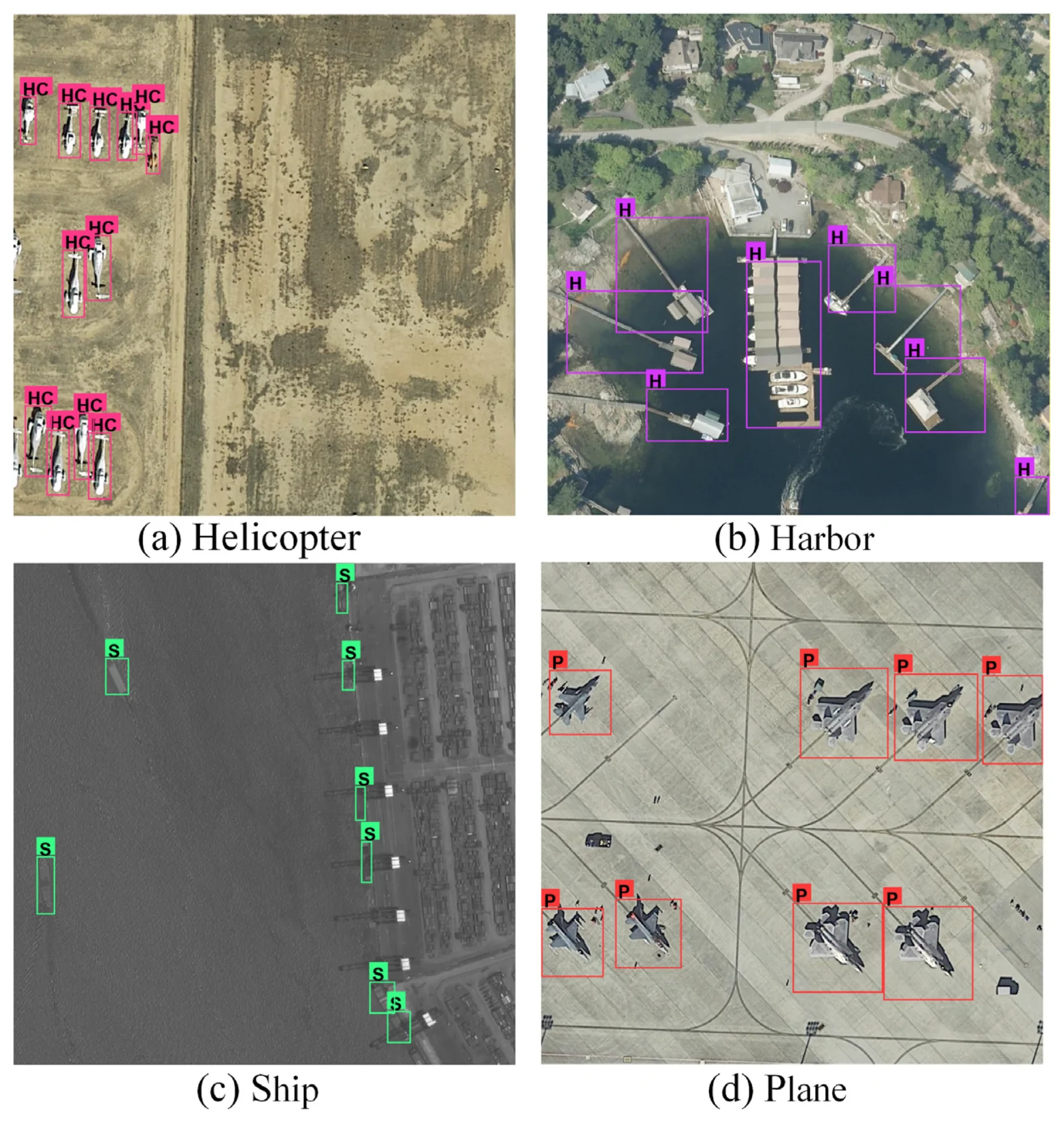
Schematic diagram of the dataset.

After the above preprocessing, a total of 21,046 image patches were obtained, including 14,732 for training, 2105 for validation, and 4209 for testing. The annotation schematic diagrams of some categories in the dataset are displayed in Figure 5.

### 3.2. Experimental Environment and Training Parameters

All experiments were implemented using Python 3.8 and the PyTorch deep learning framework. Model training and testing were conducted on an NVIDIA GeForce RTX 3090 GPU, with an Intel Core i9-12900K CPU and 128 GB of RAM. The experimental environment was configured with PyTorch 2.1.1 and CUDA 11.8. The proposed model was trained for 200 epochs with a batch size of 16 and an initial learning rate of 0.001. A cosine annealing strategy was employed to dynamically adjust the learning rate during training. Data augmentation included random scaling and spatial transformations, HSV color perturbation, horizontal flipping, Mosaic, and MixUp. The probabilities of horizontal flipping and Mosaic were both set to 0.5, while MixUp was applied with a probability of 0.5 following Mosaic. Mosaic-based augmentation was enabled during the first 70% of the training epochs. The model was optimized using SGD with a momentum of 0.937 and a weight decay of 5 × 10^−4^, and the random seed was fixed at 11. YOLOv7 pretrained weights were used for model initialization, and no early-stopping strategy was adopted. For the four-scale GRACE-YOLOv7 architecture, 12 anchors were assigned to four detection scales with strides of 32, 16, 8, and 4, and three anchors were used at each scale. For quantitative evaluation, a confidence threshold of 0.001 was used to retain candidate detections for AP calculation, and the NMS IoU threshold was set to 0.5. Precision, Recall, and F1 were calculated at a score threshold of 0.5.

### 3.3. Evaluation Metrics

To comprehensively evaluate the object detection performance of the models, Precision (P), Recall (R), F1-score, mAP0.5, and mAP0.75 were adopted as the main evaluation metrics. The Intersection over Union (IoU) was first used to quantify the spatial overlap between a predicted bounding box and its corresponding ground-truth box, which is defined as follows:

(21) $$IoU=\frac{\left|B_{p}\cap B_{g}\right|}{\left|B_{p}\cup B_{g}\right|}$$

where $B_{p}$ and $B_{g}$ denote the predicted and ground-truth bounding boxes, respectively. A prediction is regarded as a true positive (TP) when its predicted class is consistent with the ground-truth class and its IoU exceeds the specified threshold. Predictions that fail to correctly match a ground-truth object are counted as false positives (FP), whereas ground-truth objects that are not correctly detected are regarded as false negatives (FN).

Precision measures the proportion of correctly detected objects among all predicted objects, while Recall represents the proportion of ground-truth objects successfully detected by the model. They are calculated as follows:

(22) $$P=\frac{TP}{TP+FP}$$

(23) $$R=\frac{TP}{TP+FN}$$

The F1-score is the harmonic mean of Precision and Recall and provides a balanced measure of detection accuracy and completeness:

(24) $$F1=\frac{2PR}{P+R}$$

In this study, an IoU threshold of 0.5 was used for the calculation of Precision, Recall, and F1-score. For the multi-class detection task, these metrics were first calculated independently for each object category and then averaged over all 15 categories to obtain the overall Precision, Recall, and F1-score of each model.

By varying the confidence threshold, different pairs of Precision and Recall values can be obtained to construct the Precision-Recall (P-R) curve. The Average Precision (AP) for each object category is determined by the area under its P–R curve:

(25) $$AP_{i}=\int_{0}^{1}P_{i}\left(R\right)dR$$

For the multi-class detection task on DOTA-v1.0, AP is calculated independently for each object category, and the mean Average Precision (mAP) is obtained by averaging the AP values over all categories:

(26) $$mAP=\frac{1}{C}\sum_{i=1}^{C}AP_{i}$$

where C denotes the total number of object categories, and C is 15 in this study. Accordingly, mAP0.5 and mAP0.75 represent the mean AP values calculated at IoU thresholds of 0.5 and 0.75, respectively. The mAP0.5 mainly reflects the overall detection capability of the model, whereas mAP0.75 imposes a stricter localization criterion and therefore provides a more rigorous assessment of bounding-box localization accuracy. Higher values of these metrics indicate better overall detection performance.

### 3.4. Comparative Methods

To evaluate the effectiveness of the proposed method, ten representative object detection models were selected for comparison, including SSD, Faster R-CNN, RetinaNet, YOLOv5, YOLOX, YOLOv7, YOLOv8, DCEF^2^-YOLO [25], Improved-YOLOv7 [26], and DB-YOLO [27]. These detectors cover different detection paradigms, including two-stage detection frameworks, anchor-based one-stage frameworks, and anchor-free one-stage frameworks, as well as several recent improved detection architectures. YOLOv7 was used as the baseline model of the proposed method to assess the performance improvement introduced by the proposed modifications. All comparative results were obtained by retraining and testing the corresponding models on the dataset constructed in this study.

To ensure a consistent comparison, all models used exactly the same training, validation, and test subsets constructed from the same 1024 × 1024 image patches, and each model was trained for 200 epochs. Before being fed into the networks, the same image samples were resized according to the standard input configuration of each implementation: 300 × 300 pixels for SSD, 600 × 600 pixels for Faster R-CNN and RetinaNet, and 640 × 640 pixels for the YOLO-based models. These model-specific input resolutions were retained to preserve the standard operating configurations and the corresponding accuracy–efficiency trade-offs of the original implementations. Faster R-CNN employed a ResNet-50 backbone with RPN anchor scales of [8, 16, 32] and aspect ratios of [0.5, 1, 2], while RetinaNet employed a ResNet-50 backbone with P3-P7 feature pyramid levels and nine anchors per spatial location. Except for these model-specific input and architectural configurations, the remaining experimental conditions were kept as consistent as possible.

## 4. Results and Discussion

### 4.1. Detection Performance of GRACE-YOLOv7

To evaluate the overall detection performance of GRACE-YOLOv7 in complex remote sensing scenes, quantitative experiments were conducted on the DOTA-v1.0 test set, and the results are presented in Table 1. GRACE-YOLOv7 achieved mAP0.5 and mAP0.75 values of 86.09% and 65.42%, respectively, indicating good detection performance under different IoU evaluation criteria. In particular, the model still achieved a mAP of 65.42% under the stricter IoU threshold of 0.75, demonstrating its strong object localization capability. Meanwhile, Recall and Precision reached 84.40% and 82.60%, respectively, with an F1 score of 0.8347, indicating that GRACE-YOLOv7 achieves a good balance between detection completeness and detection accuracy. Overall, the model exhibits stable and comprehensive detection performance on the DOTA-v1.0 dataset, providing a basis for the subsequent analysis of class-wise detection results.

Table 2 presents the detection performance of GRACE-YOLOv7 across the 15 object categories in the DOTA-v1.0 dataset. Overall, the model demonstrates relatively stable and balanced performance across different object categories. Among them, six categories, including basketball court, large vehicle, plane, ship, small vehicle, and tennis court, achieved AP values above 90%, reaching 91.82%, 93.79%, 97.57%, 96.66%, 90.03%, and 96.30%, respectively. Plane achieved the highest AP and Recall among all categories, with values of 97.57% and 98.19%, respectively. In contrast, the bridge and soccer ball field showed relatively lower AP values of 70.68% and 72.72%, with corresponding Recall values of 67.19% and 63.75%, indicating that these two categories remain relatively challenging to detect. In terms of Precision, the ship and tennis court achieved 90.80% and 91.21%, respectively. For the F1 score, plane, ship, and tennis court all reached 0.93, whereas the soccer ball field showed a relatively lower value of 0.67. Overall, GRACE-YOLOv7 achieves high and relatively balanced detection performance for most object categories, although certain performance differences remain among different categories.

**Figure 6:**
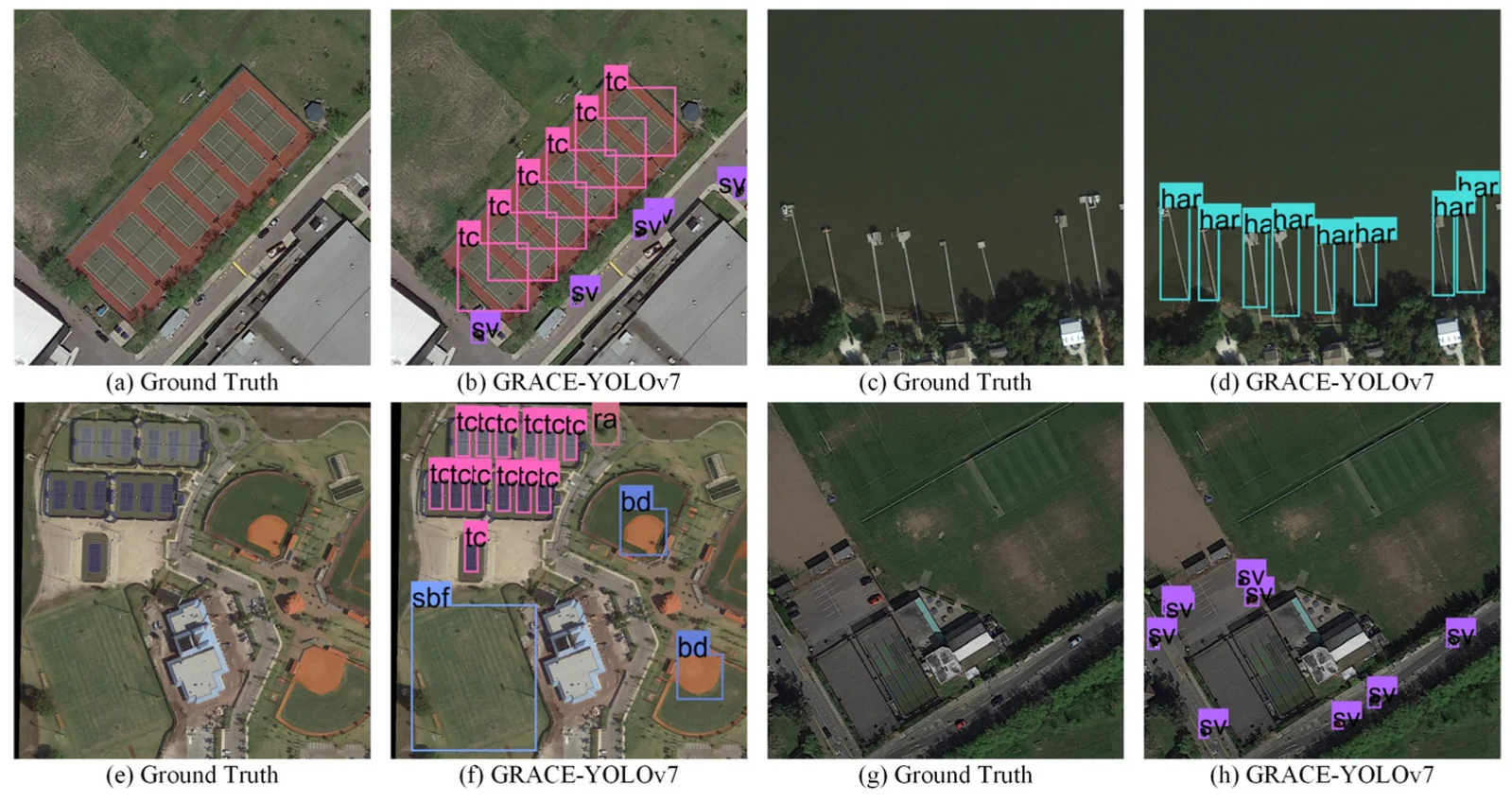
Detection results of GRACE-YOLOv7 in four representative scenarios: (**a**) a dense-object scene containing tennis courts and small vehicles; (**b**) an elongated-object scene containing harbors; (**c**) a multi-class scene containing tennis courts, soccer ball fields, and baseball diamonds; and (**d**) a small-object scene containing small vehicles.

To further visually demonstrate the detection performance of GRACE-YOLOv7 in complex remote sensing scenes, four representative test images containing dense objects, elongated objects, multiple object categories, and small objects were selected, and the corresponding detection results are shown in Figure 6. In the dense scene (Figure 6a), the image contains densely distributed tennis courts and small vehicles. GRACE-YOLOv7 is able to detect these objects relatively completely while maintaining good bounding-box localization. Figure 6b shows a harbor scene, in which the model effectively detects and localizes elongated objects with relatively large spatial extents. Figure 6c presents a multi-class scene containing tennis courts, soccer ball fields, baseball diamonds, and other object categories. GRACE-YOLOv7 is able to distinguish and localize different object categories effectively. Figure 6d shows a small-object scene dominated by small vehicles, where the model successfully identifies objects with limited spatial sizes. Overall, GRACE-YOLOv7 achieves good detection performance in the selected representative DOTA-v1.0 scenes involving dense objects, elongated objects, multiple object categories, and small objects.

### 4.2. Comparison with Other Methods

To further evaluate the detection performance of GRACE-YOLOv7, ten representative object detection models, including SSD, Faster R-CNN, RetinaNet, YOLOv5, YOLOX, YOLOv7, YOLOv8, DCEF^2^-YOLO, Improved-YOLOv7, and DB-YOLO, were selected for comparison and evaluated on the same test set. The results are presented in Table 3. Precision, Recall, and F1 were calculated at an IoU threshold of 0.5, while mAP0.5 and mAP0.75 represent the mean average precision evaluated at IoU thresholds of 0.5 and 0.75, respectively. As shown in Table 3, GRACE-YOLOv7 achieved mAP0.5, mAP0.75, Recall, and F1 values of 86.09%, 65.42%, 84.40%, and 0.8347, respectively. Among all compared models, GRACE-YOLOv7 achieved the highest mAP0.5, Recall, and F1. Compared with the baseline YOLOv7, mAP0.5 and mAP0.75 increased by 2.35 and 3.28 percentage points, respectively, while Recall increased by 2.81 percentage points and F1 improved from 0.8093 to 0.8347. GRACE-YOLOv7 achieved a higher mAP0.5 than YOLOv8 (84.16%), DCEF^2^-YOLO (55.15%), Improved-YOLOv7 (76.26%), and DB-YOLO (81.60%). In particular, GRACE-YOLOv7 outperformed YOLOv8 by 1.93 percentage points in mAP0.5 and also achieved higher Recall and F1 values. It is worth noting that the Precision of GRACE-YOLOv7 was 82.60%, slightly lower than that of RetinaNet (88.51%) and YOLOv5 (82.87%). However, GRACE-YOLOv7 achieved the highest Recall and F1, indicating that the proposed model provides better detection completeness while maintaining relatively high detection accuracy. Overall, the results demonstrate that GRACE-YOLOv7 achieves competitive performance compared with both conventional and recently improved detection models, with particular advantages in mAP0.5, Recall, and the overall balance between Precision and Recall.

**Table 2:** Class-wise detection performance of GRACE-YOLOv7 at an IoU threshold of 0.5.

	bd	bc	b	gtf	har	heli	lv	pl	ra	ship	sv	sbf	st	sp	tc	Mean
AP	88.91%	91.82%	70.68%	78.29%	89.19%	89.17%	93.79%	97.57%	65.80%	96.66%	90.03%	72.72%	87.15%	83.30%	96.30%	86.09%
F1	0.86	0.88	0.70	0.76	0.86	0.84	0.90	0.93	0.71	0.93	0.87	0.67	0.84	0.84	0.93	0.83
Recall	86.92%	91.85%	67.19%	74.65%	89.53%	89.41%	90.45%	98.19%	68.72%	94.78%	88.56%	63.75%	83.87%	82.98%	95.08%	84.40%
Precision	84.93%	83.92%	74.14%	76.81%	82.26%	78.73%	90.02%	87.86%	74.44%	90.80%	86.15%	69.86%	83.26%	84.65%	91.21%	82.60%

Note: bd = baseball diamond; bc = basketball court; b = bridge; gtf = ground track field; har = harbor; heli = helicopter; lv = large vehicle; pl = plane; ra = roundabout; ship = ship; sv = small vehicle; sbf = soccer ball field; st = storage tank; sp = swimming pool; tc = tennis court.

**Table 3:** Performance comparison of different object detection models.

Model	mAP0.5	mAP0.75	Recall	Precision	F1
SSD	44.49%	23.90%	28.69%	74.27%	0.3667
Faster-RCNN	48.73%	23.65%	58.70%	43.43%	0.4880
Retinanet	55.08%	34.20%	36.89%	88.51%	0.4747
Yolov5	78.22%	54.10%	73.17%	82.87%	0.7700
Yolox	82.68%	60.73%	79.62%	82.15%	0.8053
Yolov7	83.74%	62.14%	81.59%	80.97%	0.8093
Yolov8	84.16%	67.92%	80.71%	80.99%	0.8040
DCEF^2^-YOLO	55.15%	32.84%	38.85%	79.03%	0.4673
Improved-YOLOv7	76.26%	52.82%	69.39%	82.28%	0.7413
DB-YOLO	81.60%	57.41%	76.44%	81.22%	0.7800
GRACE-YOLOv7	86.09%	65.42%	84.40%	82.60%	0.8347

**Figure 7:**
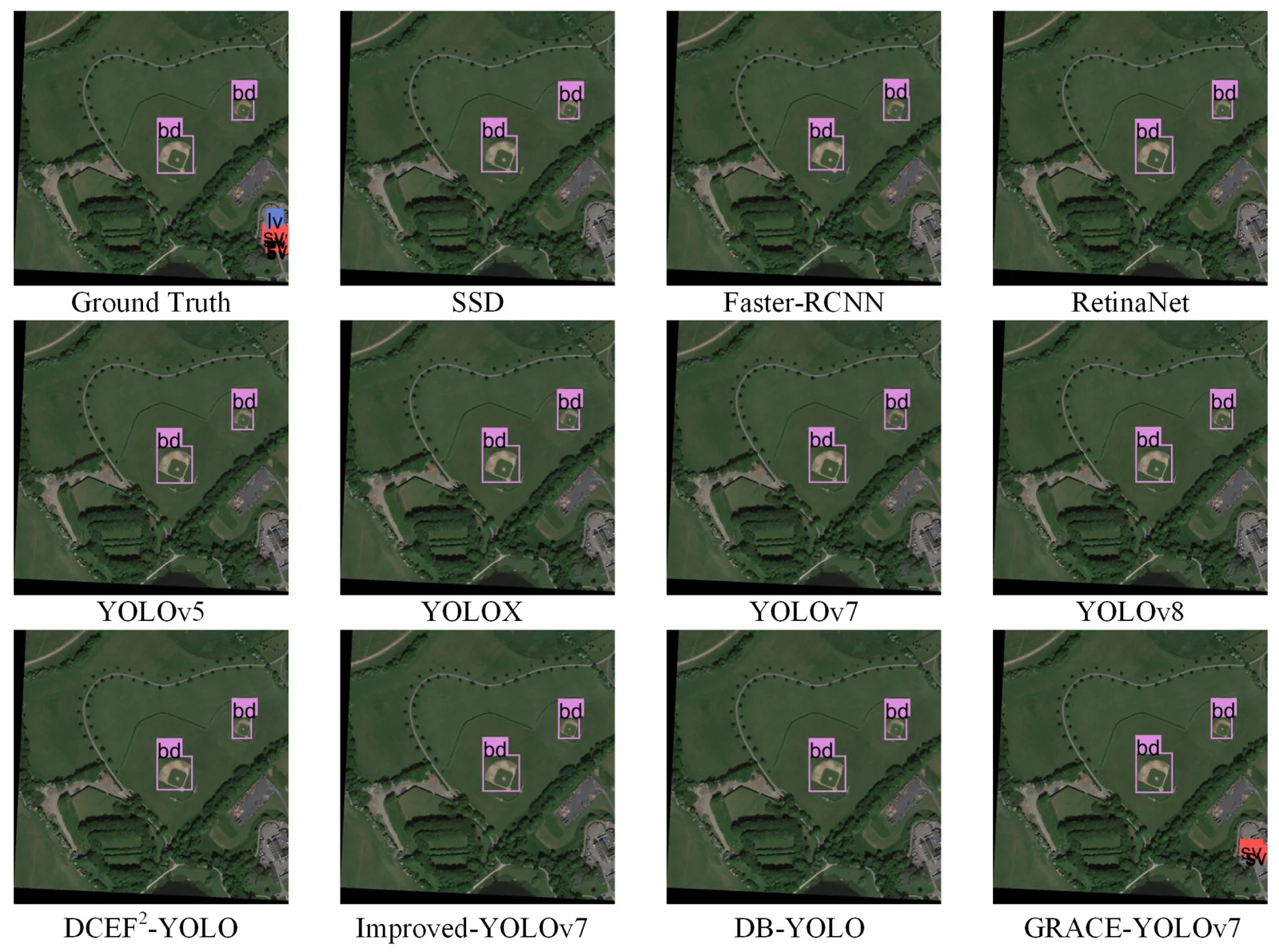
Comparison of object detection results between GRACE-YOLOv7 and other models in multi-class scenarios.

**Figure 8:**
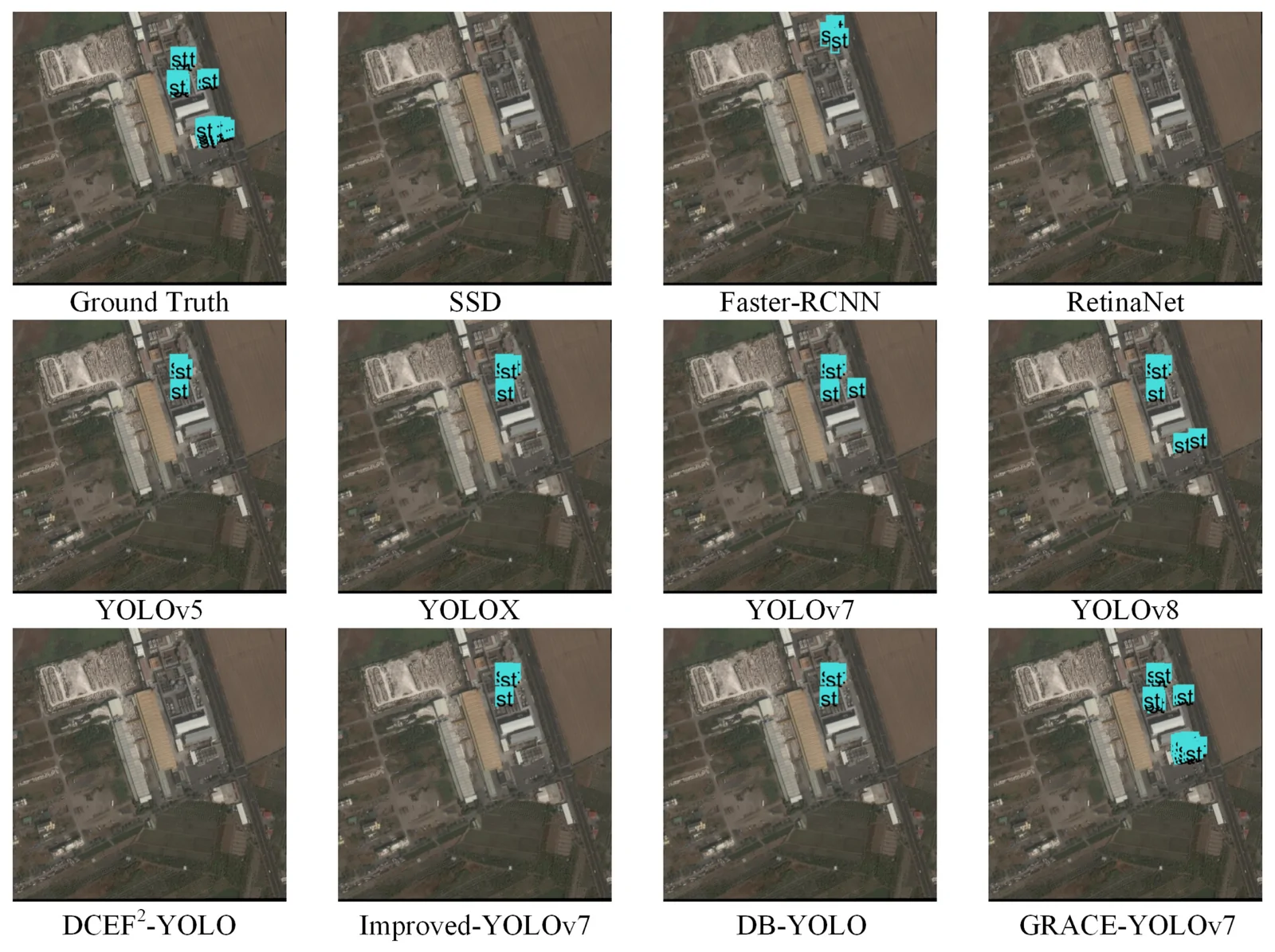
Comparison of object detection results between GRACE-YOLOv7 and other models in dense-object scenarios.

**Figure 9:**
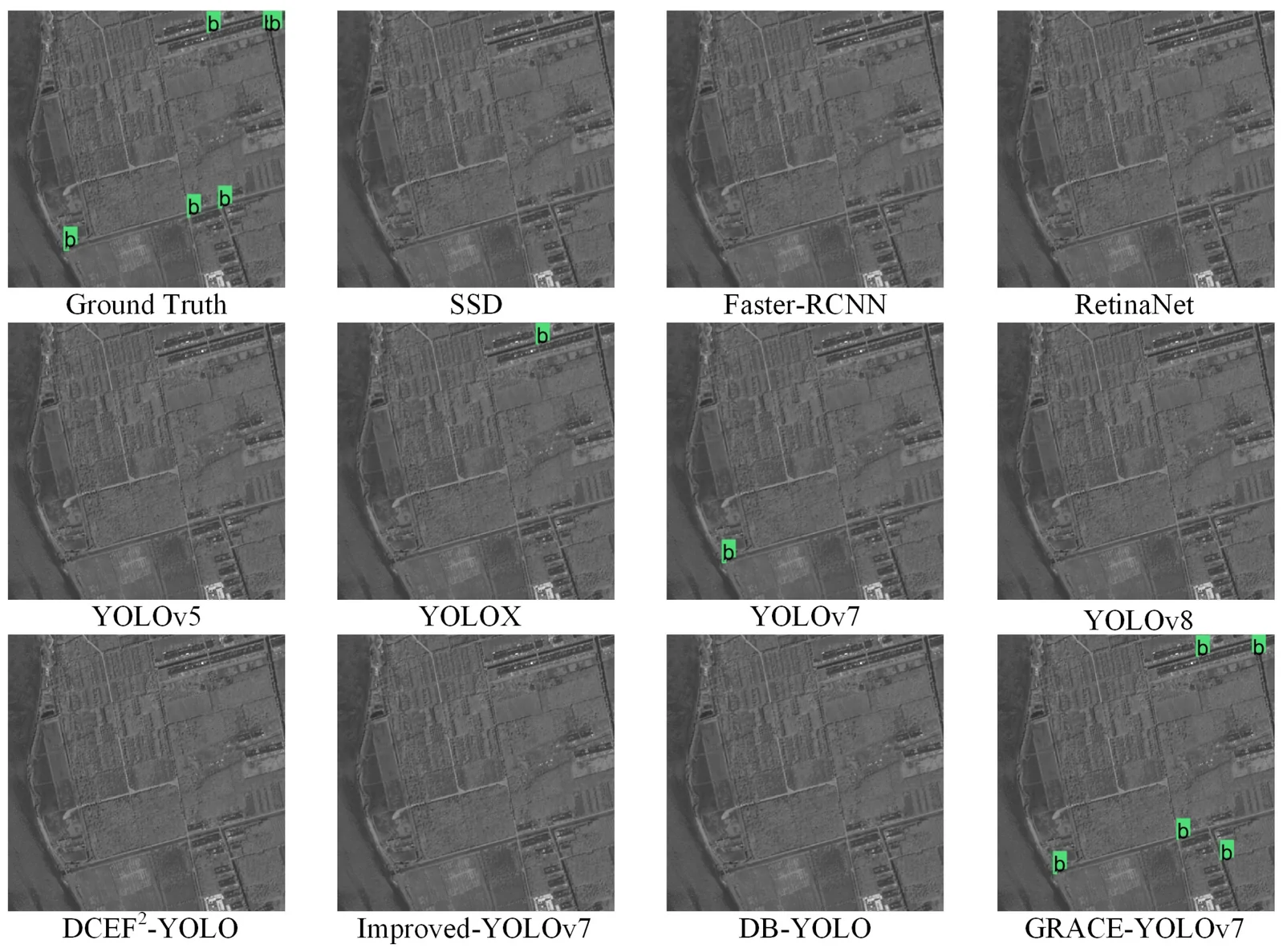
Comparison of object detection results between GRACE-YOLOv7 and other models in small-object scenarios.

**Figure 10:**
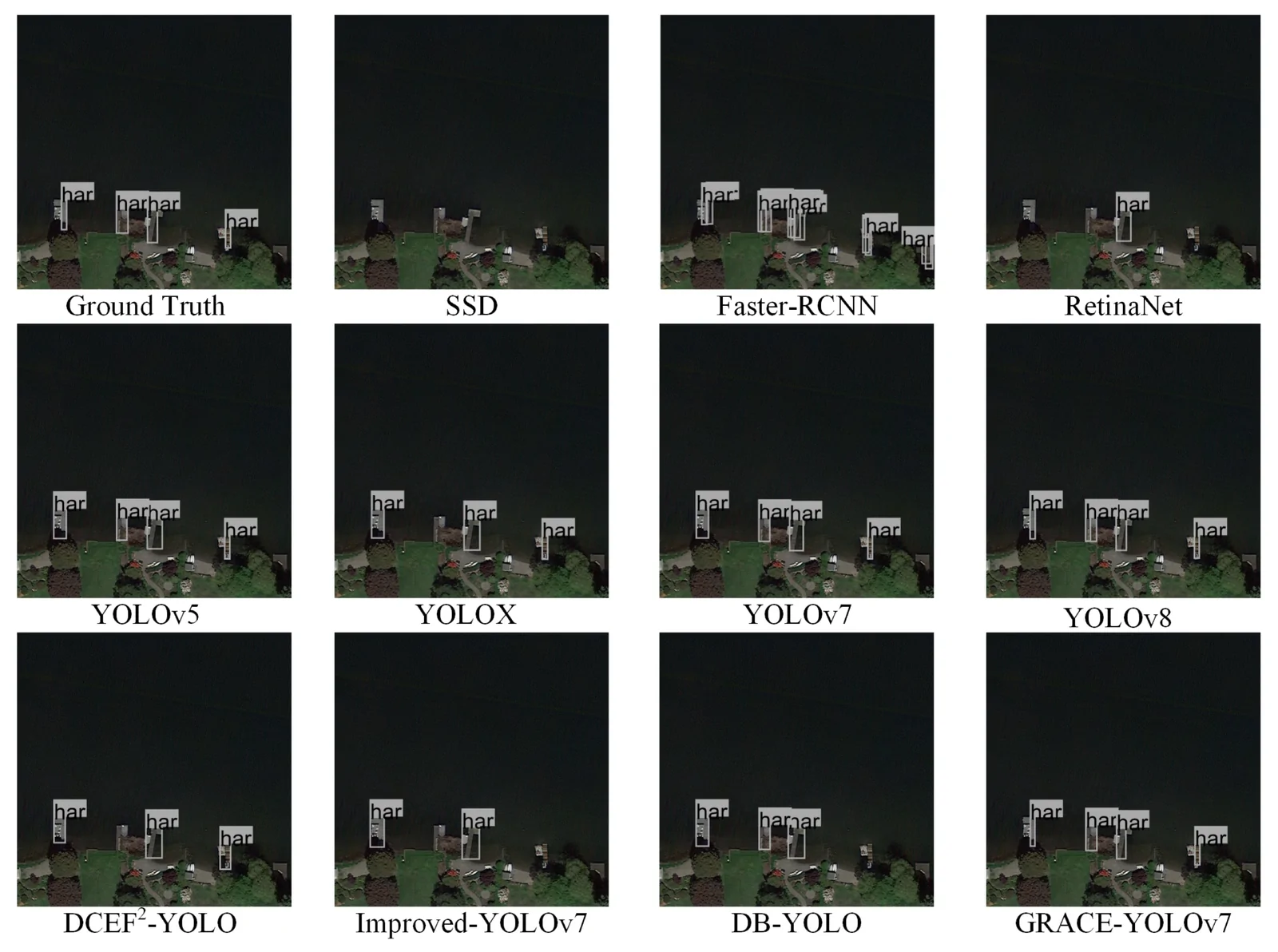
Comparison of object detection results between GRACE-YOLOv7 and other models in elongated-object scenarios.

To further visually compare the detection performance of different models in typical remote sensing scenes, four representative images were randomly selected from the DOTA-v1.0 test set, covering multi-class, dense-object, small-object, and elongated-object scenarios. The detection results of GRACE-YOLOv7 and the comparative models are shown in Figure 7, Figure 8, Figure 9 and Figure 10. All models were visualized on the same scenes, enabling a direct comparison of detection completeness and bounding-box localization performance.

Figure 7 shows the detection results in a multi-class scene containing objects of different categories and scales, including a baseball diamond, small vehicle, and large vehicle. GRACE-YOLOv7 successfully detected all baseball diamond objects and identified several small vehicles in the lower-right region of the image, whereas the other compared models failed to detect these small vehicles in this example. This result indicates that the proposed model has better recognition capability for objects of different categories and scales within the same complex scene.

Figure 8 presents a densely distributed storage tank scene. In this example, GRACE-YOLOv7 detected more storage tank objects than the other compared models, demonstrating better detection completeness for densely distributed targets. Figure 9 presents a scene containing small bridge objects, in which GRACE-YOLOv7 detected more bridge objects than the other methods, indicating better detection performance for small targets with limited spatial information.

Figure 10 presents an elongated-object scene dominated by harbor targets. GRACE-YOLOv7 accurately identified the harbor objects and achieved better bounding-box localization. Although YOLOv5 and YOLOv7 also detected the correct number of harbor objects, their predicted bounding boxes exhibited more noticeable localization deviations from the actual spatial extents of the targets compared with those of GRACE-YOLOv7. YOLOv8 also detected all harbor objects, but produced a duplicate detection for one target. Overall, the qualitative results show that, in the selected representative test scenes, GRACE-YOLOv7 achieves good comprehensive detection performance in multi-class object recognition, dense-object detection, small-object detection, and elongated-object localization.

Tables S1–S6 and S10–S13 present the AP, Recall, Precision, and F1 results of SSD, Faster R-CNN, RetinaNet, YOLOv5, YOLOX, YOLOv7, YOLOv8, DCEF^2^-YOLO, Improved-YOLOv7, and DB-YOLO for all 15 object categories in DOTA-v1.0, respectively. These metrics were calculated at an IoU threshold of 0.5. Combined with the class-wise results of GRACE-YOLOv7, the proposed model achieved noticeable performance improvements for several relatively challenging categories. Specifically, GRACE-YOLOv7 achieved an AP of 70.68% for bridge, which was 1.60 percentage points higher than the best comparative result of 69.08% achieved by YOLOv8. For the soccer ball field, the AP reached 72.72%, exceeding the best comparative result of 59.22% by 13.50 percentage points. The AP for the storage tank reached 87.15%, which was 5.58 percentage points higher than the 81.57% achieved by YOLOX. For small vehicles, GRACE-YOLOv7 achieved an AP of 90.03%, which was 0.86 percentage points higher than the 89.17% achieved by YOLOv8. These class-wise results are consistent with the qualitative observations in Figure 7, Figure 8 and Figure 9 for multi-class, dense-object, and small-object scenes, further demonstrating the good detection capability of GRACE-YOLOv7 for several challenging object categories. The mean results across the 15 categories in Tables S1–S6 and S10–S13 are consistent with the overall evaluation results reported in Table 3.

To further evaluate the computational efficiency of the compared detectors, their model complexity and inference speed were measured under a unified benchmarking protocol. All models were evaluated with a batch size of 1 using FP32 precision, with 50 warm-up iterations followed by 200 repeated forward passes. The reported latency and FPS correspond to network forward inference and exclude data loading, preprocessing, visualization, and external NMS.

As shown in Table 4, GRACE-YOLOv7 contains 38.854 M parameters and requires 122.541 GFLOPs. Compared with the original YOLOv7, the parameter count increases by only 1.584 M (4.25%), while GFLOPs increase by 16.31%. The mean inference latency increases from 14.451 to 27.878 ms/image, corresponding to a decrease in FPS from 69.20 to 35.87. Although the proposed feature-enhancement modules introduce additional runtime overhead, GRACE-YOLOv7 still has fewer parameters than YOLOv8, DB-YOLO, and Improved-YOLOv7, and requires fewer GFLOPs than Faster R-CNN, RetinaNet, YOLOv8, DB-YOLO, and Improved-YOLOv7. These results indicate that the improvement in detection accuracy is accompanied by an increase in computational cost, particularly in inference latency.

### 4.3. Ablation Study

To evaluate the effectiveness of the individual components in GRACE-YOLOv7, both progressive and independent ablation experiments were conducted using the original YOLOv7 as the baseline. In the progressive ablation experiments, ELAN-D, Bi-level Routing Attention (BRA), the high-resolution detection branch, and MPDIoU were sequentially introduced. In addition, BRA, the high-resolution detection branch, and MPDIoU were independently incorporated into the original YOLOv7 to further isolate their individual contributions. The results are presented in Table 5. The original YOLOv7 achieved mAP0.5 and mAP0.75 values of 83.74% and 62.14%, respectively. After introducing ELAN-D, these two metrics increased to 84.49% and 63.09%, corresponding to improvements of 0.75 and 0.95 percentage points over the baseline, respectively. This indicates that introducing adaptive spatial sampling during deep feature extraction can improve the overall detection performance of the model. With the further addition of BRA, mAP0.5 and mAP0.75 reached 85.03% and 63.92%, respectively, while Recall, Precision, and F1 increased to 82.71%, 81.52%, and 0.8173, respectively, indicating that introducing region-aware correlation modeling at key feature interaction positions in the Neck can further improve multi-scale feature fusion.

On this basis, after adding the high-resolution detection branch, mAP0.5 and mAP0.75 further increased to 85.76% and 64.51%, respectively. In particular, Recall increased markedly from 82.71% to 84.46%, corresponding to an improvement of 1.75 percentage points, while F1 increased from 0.8173 to 0.8300. Compared with the other stages, this modification produced the largest improvement in Recall, indicating that incorporating shallow high-resolution features and extending the detection scale can improve detection completeness. Finally, after adopting MPDIoU for bounding-box regression, the complete GRACE-YOLOv7 achieved an mAP0.5 of 86.09% and an mAP0.75 of 65.42%. Compared with the previous stage, mAP0.75 increased by 0.91 percentage points, Precision increased from 81.73% to 82.60%, while Recall remained essentially stable, changing only slightly from 84.46% to 84.40%, and F1 further increased to 0.8347. Compared with the 0.33-percentage-point increase in mAP0.5, the improvement in mAP0.75 was more pronounced, indicating that MPDIoU mainly improves bounding-box localization quality under a stricter IoU criterion. Compared with the original YOLOv7, the final GRACE-YOLOv7 improved mAP0.5, mAP0.75, Recall, and Precision by 2.35, 3.28, 2.81, and 1.63 percentage points, respectively, while F1 increased from 0.8093 to 0.8347. Overall, the model performance showed a progressive improvement as the proposed components were introduced.

The independent ablation experiments further confirm the individual contributions of the three components. When BRA was independently introduced into YOLOv7, mAP0.5 and mAP0.75 increased from 83.74% and 62.14% to 84.98% and 64.19%, respectively, while Precision increased from 80.97% to 82.70%. The independent high-resolution detection branch achieved an mAP0.5 of 85.46% and increased Recall from 81.59% to 83.67%, representing the largest Recall improvement among the independent modifications. When MPDIoU was independently adopted, mAP0.5 and mAP0.75 reached 84.86% and 64.96%, respectively. In particular, the 2.82-percentage-point improvement in mAP0.75 was more pronounced than the 1.12-percentage-point improvement in mAP0.5, further indicating that MPDIoU mainly contributes to improving bounding-box localization under a stricter IoU criterion.

Tables S6–S9 and S14–S16 further present the AP, Recall, Precision, and F1 results of YOLOv7 and the progressive and independent ablation models for all 15 object categories in DOTA-v1.0. The class-wise results show that the effects of the individual components are not entirely consistent across all categories, although the overall trend is consistent with the results reported in Table 5. For example, after introducing BRA, the AP of the soccer ball field increased from 61.49% to 71.07%, representing an improvement of 9.58 percentage points. After adding the high-resolution detection branch, the AP of the bridge increased from 65.55% to 69.64%, while that of the storage tank increased from 79.81% to 85.86%. Meanwhile, the Recall values of these two categories increased from 55.89% and 69.57% to 68.27% and 83.36%, respectively. The independent ablation results in Tables S14–S16 show similar trends. For example, independently introducing BRA increased the AP of the soccer ball field from 59.22% to 66.26%, while the high-resolution detection branch increased the AP of the bridge from 63.51% to 67.97% and its Recall from 55.29% to 62.74%. These changes are consistent with the design objectives of BRA in strengthening effective information interaction and of the high-resolution detection branch in improving feature representation for small and densely distributed objects. It should be noted that some class-specific metrics fluctuate across different ablation stages. Therefore, the contribution of each component is more appropriately evaluated based on the overall performance trend and the combined changes across multiple metrics, rather than being judged solely from the results of a single object category.

The complexity results in Table 6 further show that the different components introduce different types of computational overhead. The high-resolution detection branch mainly increases GFLOPs because of the additional high-resolution feature processing, increasing the computational cost from 105.354 to 119.883 GFLOPs when introduced independently. BRA increases the parameter count and inference latency, whereas ELAN-D introduces additional runtime cost despite its relatively limited parameter and GFLOP requirements. After combining ELAN-D, BRA, and the high-resolution detection branch, the complete inference architecture contains 38.854 M parameters and requires 122.541 GFLOPs. MPDIoU does not introduce additional inference complexity because it is used only during training.

### 4.4. Discussion

The ablation experiments, model comparisons, and qualitative results collectively indicate that the performance improvements of GRACE-YOLOv7 are associated with the combined contributions of multiple enhanced components acting at different stages of the network. First, ELAN-D introduces DCNv3 into the deep feature extraction stage of the Backbone, improving the adaptability of convolutional sampling to variations in object scale and spatial structure through content-adaptive sampling locations and feature aggregation. Previous studies have shown that deformable convolution can enhance the modeling capability of visual features for different spatial structures by dynamically adjusting the spatial sampling pattern [24]. In this study, after introducing ELAN-D, mAP0.5 and mAP0.75 increased by 0.75 and 0.95 percentage points, respectively. These performance changes are consistent with the design objective of ELAN-D to enhance deep spatial feature representation.

Second, BRA selects highly relevant spatial regions through region-level dynamic routing and further establishes fine-grained feature interactions among the selected regions, thereby reducing redundant information transmission between irrelevant regions. Related studies on BiFormer have shown that Bi-level Routing Attention can adaptively select highly correlated feature regions while maintaining effective cross-region information modeling [28]. Applications of BRA in remote sensing and other complex scenarios have also demonstrated its potential for effective spatial information modeling [30]. In this study, after introducing BRA, the overall mAP, Recall, Precision, and F1 were further improved, while the AP of categories such as soccer ball fields also showed a noticeable increase. These results indicate that region-aware correlation modeling can facilitate more effective information interaction during multi-scale feature fusion in complex remote sensing backgrounds.

The high-resolution detection branch mainly addresses the loss of spatial details of small objects during successive downsampling. Small objects in remote sensing images generally occupy only a limited number of pixels and therefore rely strongly on high-resolution spatial details. Shallow features and high-resolution detection scales play an important role in small-object detection [31]. In this study, after adding the high-resolution detection branch, Recall increased from 82.71% to 84.46%, representing the largest Recall improvement among the progressive modification stages. Meanwhile, both AP and Recall were noticeably improved for categories such as bridge and storage tank.

MPDIoU mainly acts on the bounding-box regression process. After introducing MPDIoU, mAP0.75 increased by 0.91 percentage points, which was notably larger than the corresponding 0.33-percentage-point increase in mAP0.5. Meanwhile, Precision increased by 0.87 percentage points, whereas Recall remained essentially stable. These results indicate that the main contribution of MPDIoU lies in improving bounding-box matching quality. In Figure 10, the bounding boxes predicted by GRACE-YOLOv7 for harbor objects exhibit smaller localization deviations, which is consistent with the above quantitative trend.

Overall, compared with the original YOLOv7, GRACE-YOLOv7 improved mAP0.5, mAP0.75, Recall, and Precision by 2.35, 3.28, 2.81, and 1.63 percentage points, respectively, while F1 increased from 0.8093 to 0.8347. In addition, GRACE-YOLOv7 achieved the highest mAP0.5, Recall, and F1 among all compared detection models. Although YOLOv8 achieved a higher mAP0.75 of 67.92%, GRACE-YOLOv7 maintained higher mAP0.5, Recall, and F1 values. Its Precision was also slightly lower than that of RetinaNet and YOLOv5, but the higher Recall and F1 indicate a favorable balance between missed-detection control and detection accuracy. The computational-efficiency analysis further shows that these accuracy improvements are accompanied by additional computational cost. Compared with YOLOv7, GRACE-YOLOv7 increases the parameter count from 37.270 M to 38.854 M and the computational cost from 105.354 to 122.541 GFLOPs, while the inference speed decreases from 69.20 to 35.87 FPS under the unified benchmark. Aerial remote sensing images are commonly characterized by large variations in object scale, dense spatial distributions, and pronounced changes in orientation and spatial structure, all of which increase the difficulty of object recognition and accurate localization [4]. Therefore, by jointly improving spatial feature extraction, region-aware correlation modeling, high-resolution object detection, and bounding-box regression, the proposed method enhances the overall multi-scale object detection capability of the model in complex remote sensing scenes.

### 4.5. Limitations

Despite the improved detection performance, GRACE-YOLOv7 has some limitations. The introduction of ELAN-D, BRA, and the additional detection branch increases the computational and runtime overhead compared with the original YOLOv7, indicating that further optimization is still needed to achieve a better balance between detection accuracy and inference efficiency [32]. In addition, the effectiveness of GRACE-YOLOv7 was mainly validated on the DOTA-v1.0 dataset, and its generalization across different remote sensing datasets and imaging conditions remains to be further examined. Furthermore, DOTA-v1.0 Task 2 does not provide an official small-, medium-, and large-object partition protocol, and the size-specific detection performance of the proposed model remains to be quantitatively evaluated under a consistent scale-partition criterion. Moreover, this study focuses on the DOTA-v1.0 Task 2 HBB setting. For highly oriented and densely distributed objects, such as ships and vehicles, horizontal bounding boxes may contain additional background regions and increase overlap between adjacent objects, which limits the representation of object orientation. Future work will therefore focus on developing a more lightweight architecture to reduce computational overhead while retaining its detection accuracy, conducting size-specific quantitative evaluation across different object scales, extending the proposed model to oriented bounding-box detection, and conducting cross-dataset experiments to evaluate its generalization and robustness under diverse remote sensing scenarios [33].

## 5. Conclusions

In this study, an improved YOLOv7-based detector, termed GRACE-YOLOv7, was proposed to address the challenges of substantial object-scale variations, dense spatial distributions, complex backgrounds, and inaccurate localization in remote sensing object detection. The proposed model improves the original YOLOv7 through four complementary components, including the DCNv3-enhanced ELAN-D module, Bi-level Routing Attention (BRA), an additional high-resolution detection branch, and MPDIoU-based bounding-box regression. These modifications jointly enhance spatial feature extraction, multi-scale feature fusion, small-object representation, and object localization. Experiments on the DOTA-v1.0 dataset showed that GRACE-YOLOv7 achieved mAP0.5, mAP0.75, Recall, Precision, and F1 values of 86.09%, 65.42%, 84.40%, 82.60%, and 0.8347, respectively. Compared with the baseline YOLOv7, mAP0.5, mAP0.75, Recall, and Precision increased by 2.35, 3.28, 2.81, and 1.63 percentage points, respectively, while F1 increased from 0.8093 to 0.8347. The ablation experiments verified the effectiveness and complementary contributions of the proposed components, while the comparative and qualitative results further demonstrated that GRACE-YOLOv7 provides more complete detection of small and densely distributed objects and improved localization performance in complex remote sensing scenes. Overall, GRACE-YOLOv7 effectively improves multi-scale object detection performance and provides a reliable approach for object detection in complex remote sensing imagery.

## Figures and Tables

**Table 1:** Overall detection performance of GRACE-YOLOv7.

Model	mAP0.5	mAP0.75	Recall	Precision	F1
GRACE-YOLOv7	86.09%	65.42%	84.40%	82.60%	0.8347

**Table 4:** Comparison of computational complexity and inference efficiency of different object detection models.

Model	Params (M)	GFLOPs	Mean Latency (ms/Image)	FPS
SSD	25.617	62.249	4.290	233.09
Faster R-CNN	28.419	500.370	30.483	32.81
RetinaNet	36.620	149.726	18.853	53.04
YOLOv5	7.101	16.597	8.878	112.63
YOLOX	8.943	26.787	12.209	81.91
YOLOv7	37.270	105.354	14.451	69.20
YOLOv8	43.641	165.463	15.667	63.83
DCEF^2^-YOLO	6.164	18.429	9.939	100.61
Improved-YOLOv7	65.129	130.085	20.775	48.13
DB-YOLO	56.553	160.429	19.582	51.07
GRACE-YOLOv7	38.854	122.541	27.878	35.87

**Table 5:** Ablation results of different components in GRACE-YOLOv7.

Model	mAP0.5	mAP0.75	Recall	Precision	F1
Yolov7	83.74%	62.14%	81.59%	80.97%	0.8093
Yolov7 + ELAN-D	84.49%	63.09%	81.74%	81.00%	0.8100
Yolov7 + BRA	84.98%	64.19%	81.13%	82.70%	0.8140
Yolov7 + new branch	85.46%	62.41%	83.67%	81.27%	0.8227
Yolov7 + MPDIoU	84.86%	64.96%	82.42%	82.07%	0.8193
Yolov7 + ELAN-D + BRA	85.03%	63.92%	82.71%	81.52%	0.8173
Yolov7 + ELAN-D + BRA + new branch	85.76%	64.51%	84.46%	81.73%	0.8300
GRACE-YOLOv7	86.09%	65.42%	84.40%	82.60%	0.8347

**Table 6:** Computational complexity and inference efficiency of different ablation configurations.

Model	Params (M)	GFLOPs	Mean Latency (ms/Image)	FPS
YOLOv7	37.270	105.354	14.451	69.20
YOLOv7 + ELAN-D	35.560	103.985	19.826	50.44
YOLOv7 + BRA	39.938	108.411	18.556	53.89
YOLOv7 + new branch	37.827	119.883	17.653	56.65
YOLOv7 + MPDIoU	37.270	105.354	14.451	69.20
YOLOv7 + ELAN-D + BRA	38.228	107.042	23.827	41.97
YOLOv7 + ELAN-D + BRA + new branch	38.854	122.541	27.878	35.87
GRACE-YOLOv7	38.854	122.541	27.878	35.87

Note: MPDIoU is used only during training and does not alter the inference architecture. Therefore, YOLOv7 + MPDIoU has the same inference complexity as YOLOv7, while GRACE-YOLOv7 has the same inference complexity as YOLOv7 + ELAN-D + BRA + new branch.

## Data Availability

The DOTA-v1.0 dataset used in this study is publicly available from the official DOTA website (https://captain-whu.github.io/DOTA/dataset.html accessed on 1 October 2026). The data split files and source code used in this study are available from the corresponding author upon reasonable request.

## Supplementary Materials

The following supporting information can be downloaded at: https://www.mdpi.com/article/10.3390/s26217092/s1, Table S1: Class-wise detection performance of SSD at an IoU threshold of 0.5; Table S2: Class-wise detection performance of Faster-RCNN at an IoU threshold of 0.5; Table S3: Class-wise detection performance of RetinaNet at an IoU threshold of 0.5; Table S4: Class-wise detection performance of YOLOv5 at an IoU threshold of 0.5; Table S5: Class-wise detection performance of YOLOX at an IoU threshold of 0.5; Table S6: Class-wise detection performance of YOLOv7 at an IoU threshold of 0.5; Table S7: Class-wise detection performance of YOLOv7 + ELAN-D at an IoU threshold of 0.5; Table S8: Class-wise detection performance of YOLOv7 + ELAN-D + BRA at an IoU threshold of 0.5; Table S9: Class-wise detection performance of YOLOv7 + ELAN-D + BRA + new branch at an IoU threshold of 0.5; Table S10: Class-wise detection performance of YOLOv8 at an IoU threshold of 0.5; Table S11: Class-wise detection performance of DCEF^2^-YOLO at an IoU threshold of 0.5; Table S12: Class-wise detection performance of Improved-YOLOv7 at an IoU threshold of 0.5; Table S13: Class-wise detection performance of DB-YOLO at an IoU threshold of 0.5; Table S14: Class-wise detection performance of YOLOv7 + BRA at an IoU threshold of 0.5; Table S15: Class-wise detection performance of YOLOv7 + new branch at an IoU threshold of 0.5; Table S16: Class-wise detection performance of YOLOv7 + MPDIoU at an IoU threshold of 0.5.

## Abbreviations

The following abbreviations are used in this manuscript:

BRA	Bi-level Routing Attention
DCNv3	deformable convolution v3
GRACE-YOLOv7	Geometry- and Routing-Aware Cross-scale Enhancement YOLOv7
