# Supplementary material for: GRACE-YOLOv7: An Improved Deep Learning Method for Object Detection in Remote Sensing Images
Source: Sensors (Basel). 2026 Nov 9;26(21):7092. doi: 10.3390/s26217092 (PMC13766351; doi:10.3390/s26217092)
Supplement: Supplementary file 1 [file sensors-26-07092-s001.zip › sensors-4558269-supplementary.pdf]

Table S1. Class-wise detection performance of SSD at an IoU threshold of 0.5.

	bd	bc	b	gtf	har	heli	lv	pl	ra	ship	sv	sbf	st	sp	tc	Mean
AP	63.00%	73.32%	14.29%	41.05%	57.90%	45.86%	55.47%	73.93%	26.38%	35.27%	21.04%	26.76%	18.03%	29.80%	85.17%	44.49%
F1	0.62	0.77	0.06	0.47	0.53	0.15	0.38	0.64	0.21	0.19	0.02	0.38	0.14	0.14	0.80	0.3667
Recall	48.60%	72.53%	3.00%	38.73%	40.97%	8.05%	24.79%	51.16%	12.31%	10.69%	1.07%	30.63%	7.57%	7.85%	72.41%	28.69%
Precision	86.67%	81.25%	75.76%	61.11%	73.65%	73.08%	84.23%	83.80%	77.42%	68.73%	59.15%	50.00%	72.63%	76.92%	89.70%	74.27%

Table S2. Class-wise detection performance of Faster-RCNN at an IoU threshold of 0.5.

	bd	bc	b	gtf	har	heli	lv	pl	ra	ship	sv	sbf	st	sp	tc	Mean
AP	77.35%	81.18%	23.28%	52.62%	63.41%	29.61%	50.97%	77.81%	44.68%	24.60%	17.52%	39.94%	21.46%	38.97%	87.50%	48.73%
F1	0.60	0.64	0.29	0.46	0.59	0.44	0.57	0.68	0.40	0.38	0.34	0.38	0.33	0.42	0.80	0.4880
Recall	83.18%	83.26%	38.34%	69.01%	73.40%	45.76%	56.63%	85.92%	62.05%	31.79%	31.10%	47.50%	28.75%	55.10%	88.71%	58.70%
Precision	46.35%	52.15%	22.85%	34.03%	48.70%	41.54%	57.86%	56.83%	29.30%	47.64%	36.65%	31.40%	39.26%	34.48%	72.47%	43.43%

Table S3. Class-wise detection performance of RetinaNet at an IoU threshold of 0.5.

	bd	bc	b	gtf	har	heli	lv	pl	ra	ship	sv	sbf	st	sp	tc	Mean
AP	71.67%	74.40%	25.61%	59.68%	65.19%	75.35%	54.94%	86.84%	52.36%	41.82%	23.72%	31.42%	25.77%	49.26%	88.15%	55.08%
F1	0.64	0.75	0.18	0.61	0.63	0.64	0.55	0.84	0.19	0.35	0.01	0.34	0.29	0.23	0.87	0.4747
Recall	48.60%	65.24%	9.86%	45.77%	50.72%	50.00%	40.92%	76.00%	10.77%	21.67%	0.64%	20.62%	17.41%	13.35%	81.75%	36.89%
Precision	92.86%	88.89%	83.67%	90.28%	82.01%	90.77%	81.74%	94.71%	95.45%	91.89%	65.79%	89.19%	94.82%	93.58%	91.98%	88.51%

Table S4. Class-wise detection performance of YOLOv5 at an IoU threshold of 0.5.

	bd	bc	b	gtf	har	heli	lv	pl	ra	ship	sv	sbf	st	sp	tc	Mean
AP	85.49%	83.54%	53.73%	66.71%	87.20%	86.56%	88.21%	96.80%	68.73%	91.31%	83.93%	35.28%	71.12%	80.06%	94.64%	78.22%
F1	0.81	0.84	0.51	0.65	0.85	0.84	0.85	0.92	0.71	0.88	0.82	0.42	0.75	0.78	0.92	0.7700
Recall	78.97%	83.69%	38.46%	61.97%	85.57%	87.71%	80.64%	95.95%	63.08%	85.87%	76.98%	31.87%	63.64%	70.81%	92.36%	73.17%
Precision	82.84%	83.69%	74.59%	68.75%	83.65%	80.86%	90.00%	88.97%	81.46%	90.74%	87.74%	60.71%	91.68%	86.01%	91.35%	82.87%

Table S5. Class-wise detection performance of YOLOX at an IoU threshold of 0.5.

	bd	bc	b	gtf	har	heli	lv	pl	ra	ship	sv	sbf	st	sp	tc	Mean
AP	85.49%	87.04%	63.31%	77.22%	89.35%	90.47%	92.04%	97.21%	70.01%	95.62%	88.51%	43.06%	81.57%	84.21%	95.08%	82.68%
F1	0.83	0.86	0.64	0.75	0.86	0.84	0.88	0.93	0.70	0.92	0.85	0.46	0.81	0.82	0.93	0.8053
Recall	82.24%	88.84%	56.13%	80.28%	88.68%	87.71%	87.34%	97.68%	63.08%	92.79%	83.94%	38.75%	72.57%	80.24%	93.97%	79.62%
Precision	84.21%	83.13%	75.69%	69.94%	82.95%	80.86%	87.89%	88.15%	79.87%	90.64%	85.97%	56.88%	90.43%	83.86%	91.71%	82.15%

Table S6. Class-wise detection performance of YOLOv7 at an IoU threshold of 0.5.

	bd	bc	b	gtf	har	heli	lv	pl	ra	ship	sv	sbf	st	sp	tc	Mean
AP	87.67%	87.41%	63.51%	81.50%	89.69%	88.16%	92.68%	96.74%	65.77%	96.29%	89.03%	59.22%	80.29%	82.51%	95.60%	83.74%
F1	0.82	0.85	0.64	0.76	0.86	0.85	0.90	0.92	0.67	0.92	0.86	0.54	0.80	0.82	0.93	0.8093
Recall	84.58%	90.13%	55.29%	85.92%	90.45%	90.25%	89.43%	97.89%	61.54%	94.28%	85.45%	53.12%	71.43%	79.45%	94.65%	81.59%
Precision	78.70%	80.15%	74.68%	67.78%	82.56%	80.99%	89.63%	86.71%	74.53%	90.70%	87.36%	55.19%	89.63%	85.01%	90.87%	80.97%

Table S7. Class-wise detection performance of YOLOv7+ELAN-D at an IoU threshold of 0.5.

	bd	bc	b	gtf	har	heli	lv	pl	ra	ship	sv	sbf	st	sp	tc	Mean
AP	90.14%	87.31%	63.47%	79.28%	90.26%	90.54%	92.65%	96.83%	70.50%	96.19%	90.03%	61.49%	80.67%	82.24%	95.75%	84.49%
F1	0.86	0.85	0.64	0.76	0.86	0.85	0.89	0.92	0.73	0.92	0.87	0.46	0.80	0.81	0.93	0.8100
Recall	89.25%	88.41%	55.17%	87.32%	90.35%	88.98%	88.94%	97.84%	71.28%	93.94%	87.40%	41.25%	72.46%	78.93%	94.65%	81.74%
Precision	82.68%	82.07%	75.25%	67.76%	82.64%	80.77%	89.67%	86.46%	74.73%	90.64%	86.40%	52.38%	88.68%	83.75%	91.09%	81.00%

Table S8. Class-wise detection performance of YOLOv7+ELAN-D+BRA at an IoU threshold of 0.5.

	bd	bc	b	gtf	har	heli	lv	pl	ra	ship	sv	sbf	st	sp	tc	Mean
AP	88.33%	88.96%	65.55%	79.19%	89.48%	90.50%	93.29%	97.56%	68.68%	96.02%	88.28%	71.07%	79.81%	82.96%	95.77%	85.03%
F1	0.85	0.85	0.64	0.74	0.86	0.85	0.90	0.92	0.70	0.92	0.86	0.65	0.78	0.81	0.93	0.8173
Recall	86.92%	89.27%	55.89%	80.99%	88.84%	90.68%	89.55%	97.73%	66.67%	93.15%	84.97%	73.12%	69.57%	79.06%	94.23%	82.71%
Precision	82.67%	81.25%	75.12%	67.65%	84.24%	79.26%	89.63%	87.68%	74.29%	91.33%	86.94%	59.09%	89.04%	83.31%	91.36%	81.52%

Table S9. Class-wise detection performance of YOLOv7+ELAN-D+BRA+new branch at an IoU threshold of 0.5.

	bd	bc	b	gtf	har	heli	lv	pl	ra	ship	sv	sbf	st	sp	tc	Mean
AP	89.59%	90.18%	69.64%	77.56%	89.53%	89.21%	93.65%	97.74%	68.68%	96.79%	89.69%	68.67%	85.86%	83.98%	95.69%	85.76%
F1	0.86	0.88	0.70	0.74	0.86	0.84	0.90	0.92	0.71	0.93	0.87	0.67	0.82	0.83	0.92	0.8300
Recall	88.32%	90.13%	68.27%	73.24%	90.28%	90.68%	90.46%	98.16%	69.23%	94.72%	86.89%	66.25%	83.36%	82.98%	93.97%	84.46%
Precision	83.63%	85.02%	71.90%	74.29%	82.95%	78.10%	89.67%	87.45%	72.19%	90.62%	86.79%	67.52%	81.48%	83.75%	90.66%	81.73%

Table S10. Class-wise detection performance of YOLOv8 at an IoU threshold of 0.5.

	bd	bc	b	gtf	har	heli	lv	pl	ra	ship	sv	sbf	st	sp	tc	Mean
AP	90.34%	89.14%	69.08%	78.56%	91.61%	93.09%	92.60%	96.82%	70.10%	95.92%	89.17%	45.02%	81.21%	83.84%	95.90%	84.16%
F1	0.82	0.85	0.66	0.69	0.88	0.85	0.90	0.93	0.71	0.92	0.86	0.44	0.79	0.83	0.93	0.8040
Recall	83.64%	89.70%	57.69%	79.58%	90.87%	91.95%	89.49%	98.32%	65.13%	92.94%	85.04%	41.25%	70.15%	80.37%	94.48%	80.71%
Precision	81.36%	81.64%	78.05%	61.41%	85.08%	78.62%	90.54%	87.49%	77.44%	91.32%	87.10%	48.18%	90.17%	85.40%	91.08%	80.99%

Table S11. Class-wise detection performance of DCEF²-YOLO at an IoU threshold of 0.5.

	bd	bc	b	gtf	har	heli	lv	pl	ra	ship	sv	sbf	st	sp	tc	Mean
AP	76.58%	77.81%	33.71%	3.06%	77.18%	0.71%	72.53%	92.83%	46.14%	64.02%	62.40%	15.34%	42.75%	69.23%	92.94%	55.15%
F1	0.76	0.79	0.22	0.00	0.72	0.01	0.71	0.89	0.00	0.60	0.52	0.01	0.48	0.40	0.90	0.4673
Recall	65.89%	67.81%	12.86%	0.00%	60.73%	0.42%	59.06%	87.95%	0.00%	44.80%	37.29%	0.62%	32.63%	26.18%	86.50%	38.85%
Precision	88.68%	95.76%	79.85%	0.00%	89.88%	100.00%	90.22%	91.02%	0.00%	91.60%	87.08%	100.00%	91.49%	86.58%	93.32%	79.03%

Table S12. Class-wise detection performance of Improved-YOLOv7 at an IoU threshold of 0.5.

	bd	bc	b	gtf	har	heli	lv	pl	ra	ship	sv	sbf	st	sp	tc	Mean
AP	80.15%	80.65%	47.05%	60.77%	85.03%	89.53%	89.71%	96.85%	50.50%	93.75%	84.96%	36.80%	73.94%	79.51%	94.63%	76.26%
F1	0.77	0.81	0.45	0.57	0.83	0.84	0.86	0.92	0.51	0.90	0.80	0.42	0.76	0.77	0.91	0.7413
Recall	71.50%	78.11%	32.33%	48.59%	82.30%	88.56%	82.08%	95.81%	37.44%	88.52%	76.71%	30.63%	65.65%	71.73%	90.83%	69.39%
Precision	82.70%	84.26%	74.31%	67.65%	84.40%	79.77%	89.30%	88.32%	78.49%	90.99%	84.53%	65.33%	89.30%	83.54%	91.30%	82.28%

Table S13. Class-wise detection performance of DB-YOLO at an IoU threshold of 0.5.

	bd	bc	b	gtf	har	heli	lv	pl	ra	ship	sv	sbf	st	sp	tc	Mean
AP	84.32%	85.42%	56.32%	75.59%	87.19%	89.32%	91.68%	96.94%	67.59%	95.41%	87.50%	57.76%	75.13%	77.98%	95.89%	81.60%
F1	0.80	0.83	0.52	0.71	0.85	0.83	0.88	0.92	0.67	0.91	0.84	0.50	0.76	0.75	0.93	0.7800
Recall	79.44%	84.55%	40.14%	79.58%	85.47%	89.83%	87.23%	96.84%	58.46%	90.45%	83.60%	44.38%	65.69%	66.88%	94.06%	76.44%
Precision	80.57%	81.74%	75.06%	63.48%	83.83%	77.37%	88.67%	88.19%	79.17%	91.31%	85.02%	57.26%	89.53%	86.03%	91.04%	81.22%

Table S14. Class-wise detection performance of YOLOv7+BRA at an IoU threshold of 0.5.

	bd	bc	b	gtf	har	heli	lv	pl	ra	ship	sv	sbf	st	sp	tc	Mean
AP	89.16%	90.90%	64.91%	80.70%	88.73%	90.11%	93.12%	97.35%	70.68%	96.13%	88.57%	66.26%	80.39%	82.24%	95.47%	84.98%
F1	0.84	0.87	0.61	0.77	0.85	0.86	0.89	0.92	0.70	0.92	0.85	0.60	0.79	0.81	0.93	0.8140
Recall	84.11%	90.99%	50.96%	83.10%	87.89%	90.25%	88.43%	97.49%	64.62%	93.17%	83.18%	60.00%	70.99%	77.09%	94.65%	81.13%
Precision	84.11%	84.13%	77.23%	71.08%	83.21%	81.30%	90.33%	87.80%	76.83%	91.33%	87.72%	60.38%	89.24%	84.87%	90.95%	82.70%

Table S15. Class-wise detection performance of YOLOv7+new branch at an IoU threshold of 0.5.

	bd	bc	b	gtf	har	heli	lv	pl	ra	ship	sv	sbf	st	sp	tc	Mean
AP	91.41%	86.93%	67.97%	76.02%	89.80%	90.73%	93.44%	97.48%	69.66%	96.67%	89.36%	66.66%	85.90%	83.50%	96.30%	85.46%
F1	0.84	0.86	0.68	0.76	0.86	0.83	0.90	0.92	0.70	0.93	0.87	0.60	0.83	0.83	0.93	0.8227
Recall	86.92%	88.84%	62.74%	80.99%	90.84%	91.53%	90.32%	98.19%	67.18%	95.52%	87.37%	56.25%	81.75%	81.68%	94.91%	83.67%
Precision	81.58%	84.15%	75.11%	70.99%	81.94%	75.79%	89.07%	86.17%	74.01%	90.20%	86.00%	64.29%	85.11%	83.87%	90.75%	81.27%

Table S16. Class-wise detection performance of YOLOv7+MPDIoU at an IoU threshold of 0.5.

	bd	bc	b	gtf	har	heli	lv	pl	ra	ship	sv	sbf	st	sp	tc	Mean
AP	88.91%	88.08%	64.29%	80.66%	90.57%	90.70%	92.85%	97.08%	69.36%	96.51%	89.23%	63.84%	81.43%	83.35%	96.06%	84.86%
F1	0.84	0.86	0.63	0.73	0.88	0.87	0.90	0.92	0.71	0.93	0.87	0.60	0.79	0.83	0.93	0.8193
Recall	87.85%	87.98%	53.61%	83.80%	90.80%	89.83%	89.29%	98.00%	65.13%	94.31%	85.67%	65.00%	70.48%	79.97%	94.57%	82.42%
Precision	81.39%	83.33%	75.72%	64.32%	84.73%	83.79%	90.64%	86.73%	77.44%	91.33%	88.56%	56.52%	89.96%	85.45%	91.09%	82.07%

Note: bd = baseball diamond; bc = basketball court; b = bridge; gtf = ground track field; har = harbor; heli = helicopter; lv = large vehicle; pl = plane; ra = roundabout; ship = ship; sv = small vehicle; sbf = soccer ball field; st = storage tank; sp = swimming pool; tc = tennis court.
